# Range-wide genetic structure in the thorn-tailed rayadito suggests limited gene flow towards peripheral populations

**DOI:** 10.1038/s41598-020-66450-7

**Published:** 2020-06-10

**Authors:** Esteban Botero-Delgadillo, Veronica Quirici, Yanina Poblete, Matías Acevedo, Élfego Cuevas, Camila Bravo, Margherita Cragnolini, Ricardo Rozzi, Elie Poulin, Jakob C. Mueller, Bart Kempenaers, Rodrigo A. Vásquez

**Affiliations:** 1 Instituto de Ecología y Biodiversidad, Departamento de Ciencias Ecológicas, Facultad de Ciencias, Universidad de Chile, Santiago, Chile; 2 Department of Behavioural Ecology and Evolutionary Genetics, Max Plank Institute for Ornithology, Seewiesen, Germany; 3 SELVA: Research for conservation in the Neotropics, Bogotá, Colombia; 4 Departamento de Ecología y Biodiversidad, Facultad de Ecología y Recursos Naturales, Universidad Andrés Bello, Santiago, Chile; 5 Centro de investigación para la sustentabilidad, Universidad Andrés Bello, Santiago, Chile; 6 Instituto de Ciencias Naturales, Universidad de las Américas, Santiago, Chile; 7 Programa de Magister en Áreas Silvestres y Conservación de la Naturaleza, Facultad de Ciencias Forestales y Conservación de la Naturaleza, Universidad de Chile, Santiago, Chile; 8 Doctorado en Medicina de la Conservación, Facultad de Ecología y Recursos Naturales, Universidad Andrés Bello, Santiago, Chile; 9 Programa de Conservación Biocultural Sub-Antártica, Parque Etnobotánico Omora, Universidad de Magallanes & Instituto de Ecología y Biodiversidad, Santiago, Chile; 10 Sub-Antarctic Biocultural Conservation Program, Department of Philosophy and Religion & Department of Biological Sciences, University of North Texas, Denton, TX USA

**Keywords:** Population genetics, Behavioural ecology, Molecular ecology, Population dynamics

## Abstract

Understanding the population genetic consequences of habitat heterogeneity requires assessing whether patterns of gene flow correspond to landscape configuration. Studies of the genetic structure of populations are still scarce for Neotropical forest birds. We assessed range-wide genetic structure and contemporary gene flow in the thorn-tailed rayadito (*Aphrastura spinicauda*), a passerine bird inhabiting the temperate forests of South America. We used 12 microsatellite loci to genotype 582 individuals from eight localities across a large latitudinal range (30°S–56°S). Using population structure metrics, multivariate analyses, clustering algorithms, and Bayesian methods, we found evidence for moderately low regional genetic structure and reduced gene flow towards the range margins. Genetic differentiation increased with geographic distance, particularly in the southern part of the species’ distribution where forests are continuously distributed. Populations in the north seem to experience limited gene flow likely due to forest discontinuity, and may comprise a demographically independent unit. The southernmost population, on the other hand, is genetically depauperate and different from all other populations. Different analytical approaches support the presence of three to five genetic clusters. We hypothesize that the genetic structure of the species follows a hierarchical clustered pattern.

## Introduction

Investigating how genetic variation is distributed across a species’ geographic range is fundamental to identify the factors contributing to demographic and population structure^1^. Studies of range-wide genetic structure not only provide information about dispersal rates and population connectivity, but also allow a better understanding of how contemporary population dynamics are linked to spatial and temporal environmental variation^2^. In species with restricted dispersal, genetic variation is often found to vary continuously across space, such that genetic differentiation increases with geographic distance –isolation by distance (IBD)^3–5^. However, distinct landscape features can act as barriers to dispersal and may have a profound impact on gene flow and population dynamics –isolation by resistance (IBR)^6^. As limited dispersal and physical and environmental barriers promote the isolation of local populations, they can ultimately lead to genetic differentiation and divergence or to local extinctions^7,8^.

The patterns of spatial distribution of neutral genetic variation vary among organisms depending on their dispersal capacity and their level of ecological specialization^9^. The vagility of organisms like birds allows them to overcome many potential barriers to gene flow. Consequently, birds exhibit lower levels of genetic structure compared to other vertebrates^10^. On the other hand, behavioral barriers to dispersal such as philopatry^11^ can prevent gene flow and promote genetic differentiation^12^. In fact, genetic differentiation has been documented even at local scales in several bird species^13–15^. Furthermore, it has been shown that variation in bird dispersal capacity is associated with differences in ecological traits^16^. For instance, dispersal propensity is higher in frugivorous and canopy birds compared to insectivorous species or those inhabiting the forest understory^16,17^. Likewise, forest interior birds are less prone to cross open areas than those that prefer forest edges or early successional habitats^18^.

Spatial patterns of genetic variation and divergence not only depend on ecological and life-history differences among species, but also on the way a species responds to landscape heterogeneity and habitat loss. Habitat specialization in birds seems to be a good predictor of genetic response to habitat loss and fragmentation^19,20^. Among Neotropical species, for example, the demographic and genetic consequences of forest fragmentation appear to be more severe for forest specialists^16,19,21^. Given the accelerated rates of deforestation in the Neotropics and the lack of information on spatial patterns of genetic diversity for several species, investigating range-wide genetic structure is crucial to determine the factors affecting gene flow and to identify local populations with unique evolutionary trajectories^22^. This is essential to inform conservation planning and to predict the genetic consequences of rapid environmental change.

**Figure 1:**
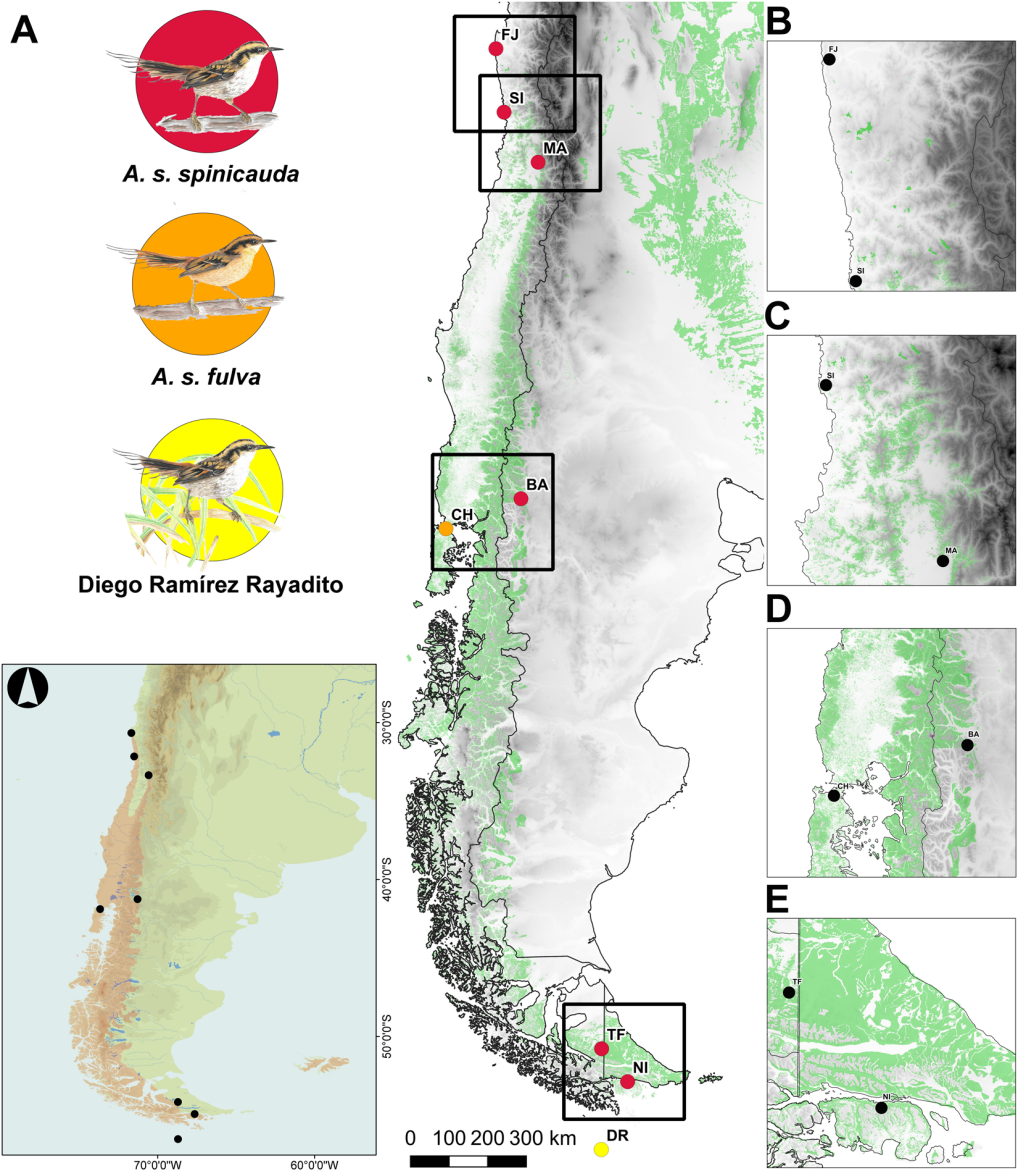
Sampled localities and study populations. (**A**) Eight localities were sampled across the breeding range of thorn-tailed rayadito in Chile and Argentina (light red on the map). Colors correspond to each taxon sampled: *Aphrastura spinicauda spinicauda* (nominate subspecies), *A. s. fulva*, and the rayadito from the Diego Ramírez Archipelago, presumably a different taxon. Shown is the elevational profile (in greyscale) and the native forest remnants across Chile and Argentina (in green). Areas enclosed by quadrats are detailed in panels (**B**–**E**). Maps were created in the free software QGIS 3.8.2 (https://qgis.org) using geographic layers from the SRTM 90 m Digital Elevation Database v4.1 (https://cgiarcsi.community/data/srtm-90m-digital-elevation-database-v4-1/), Sistema de Información Territorial (SIT CONAF; http://sit.conaf.cl), and Instituto Geográfico Nacional (IGN; https://www.ign.gob.ar). Illustrations by Priscila Escobar Gimpel.

Here we assessed spatial patterns of genetic diversity, range-wide genetic structure, and contemporary gene flow in a small forest passerine bird of South America. We studied the thorn-tailed rayadito (*Aphrastura spinicauda*), a secondary-cavity nester found in south-temperate forests distributed along a large latitudinal range (~30°S–56°S) in Chile and western Argentina^23^ (Fig. 1). As other members of the family Furnariidae, the thorn-tailed rayadito is a resident and relatively sedentary species^23,24^. As is typical for socially monogamous songbirds, male thorn-tailed rayaditos are more philopatric and show within-population genetic structure due to locally restricted movements, while females are the dispersing sex^25,26^. Despite its non-migratory habits and its rather philopatric behavior, this bird has successfully colonized oceanic islands located ~35–100 km off the continent^23^. A recent study provided genetic evidence of recurrent gene flow between eight populations across its breeding range^27^, although the sample size per population was limited (9–21 individuals). Quantitative information on dispersal rates is currently lacking, and it remains unclear whether contemporary gene flow is affected by forest fragmentation.

We genotyped 582 individuals from eight different populations of thorn-tailed rayadito at 12 polymorphic microsatellite loci to (i) explore genetic diversity and range-wide spatial structure and (ii) quantify contemporary gene flow. To this end, we combined population structure metrics, multivariate analyses, clustering algorithms, and Bayesian methods. We describe how genetic diversity varies along the extensive latitudinal gradient, encompassing most of the species’ distribution, and relate the observed patterns to the availability of forested habitats. We also discuss some conservation and taxonomic implications of our results.

## Methods

### Study species and populations

The thorn-tailed rayadito is a forest specialist that breeds in tree cavities in old-growth forests^28,29^. Rayaditos also use second-growth forests and exotic pine plantations as breeding habitats when nestboxes are provided^30,31^. Nest-site supplementation experiments suggest that populations in these atypical habitats are limited by nest site availability^30,32^. Population responses to nest-site supplementation further suggest that rayaditos are sensitive to forest fragmentation^29,33^. Birds inhabiting fragmented landscapes seldom cross open areas between forest patches, and flights through non-forest habitats usually are <300 m^34^.

Three subspecies of thorn-tailed rayadito are recognized based on morphological differences: *A. s. spinicauda*, the most widespread form that is distributed across mainland Chile and Argentina and is also present on some islands along the Chilean coast; *A. s. bullocki*, endemic to Mocha Island; and *A. s. fulva*, restricted to Chiloé Island and the Chonos islands^23^. A population occupying the remote islands of the Diego Ramírez Archipelago has long been considered as *A. s. spinicauda*^23^, but behavioral, morphological and genetic evidence suggests that it might be a different taxon (Rozzi *et al*., unpublished).

We collected blood samples of birds from eight localities across the distribution of thorn-tailed rayadito, including *A. s. spinicauda*, *A. s. fulva*, and the population from Diego Ramírez (Fig. 1, Table 1). The two northernmost study sites, Fray Jorge National Park (FJ) and Cerro Santa Inés (SI), are located in the Chilean semiarid region, where relicts of Valdivian temperate forests occur on coastal mountaintops (500–700 masl) and persist due to oceanic fog-water inputs^34,35^. Once a continuous forest, these relicts have been subjected to a gradual process of fragmentation and isolation due to climatic changes, becoming restricted to the coastal mountain range during the Quaternary^36,37^. FJ consists of several forest fragments that extend for ~2.4 km^2^ in a shrub-dominated matrix^38^ and constitutes the northerly margin of the species’ range (Fig. 1). Smaller remnants are found along watersheds and mountaintops farther south, including the 53 ha relict in SI^37^, located ~170 km from FJ. The third study site is Cerro Manquehue (MA), in the metropolitan district in central Chile, in which sclerophyllous forests are the main habitat of rayaditos^24^. The distribution of wooden habitats in this region is more continuous (Fig. 1), but humid forests where rayaditos mostly occur have been extensively degraded due to rapid urban development^24,39^. The remaining study sites are located below 41° S, where native forests are more continuously distributed (Fig. 1). Despite widespread forestry plantations in central-south Chile, the proportion of old-growth humid temperate forests increases towards the south (Supplementary Fig. S1). The study sites at Bariloche (BA) and Chiloé Island (CH) are in the center of the species’ range, located on the eastern and western side of the Andes, respectively (Fig. 1). Tierra del Fuego (TF) is the southernmost mainland locality that was sampled. The study site at Navarino Island (NI) is separated from the continent by the 6 km wide Beagle Channel (Fig. 1). Samples from the population in Diego Ramírez (DR) were collected at Gonzalo Island, ~100 km southwest of Cape Horn. The archipelago lacks arborescent vegetation and rayaditos nest at ground level in crevices under tussocks of the grass *Poa flavellata*^23^.

**Table 1:** Locations, sample size (females and males), genetic diversity statistics and demographic parameters for eight populations of thorn-tailed rayadito.

Locality	n (f, m)	Parameter							
		*N*_*a*_	*N*_a_*	*N*_*p*_	H_O_	H_e_	F_IS_	*N*_e_**	Demography^
FJ	183 (95, 88)	7.42 ± 0.88	4.88 ± 0.47	0.08 ± 0.08	0.67 ± 0.05	0.68 ± 0.05	0.005	121 (100-148)/∞ (42-∞)	Bott/Equi/**Equi**
SI	10 (5, 5)	5.00 ± 0.46	4.88 ± 0.44	0.08 ± 0.08	0.75 ± 0.04	0.71 ± 0.03	−0.128	—	—
MA	46 (22, 24)	8.75 ± 1.06	6.25 ± 0.65	0.25 ± 0.13	0.72 ± 0.06	0.73 ± 0.07	0.012	36 (30-44)/∞ (32-∞)	Equi/Equi/**Equi**
BA	61 (28, 33)	13.50 ± 2.04	7.21 ± 0.84	1.33 ± 0.59	0.75 ± 0.05	0.77 ± 0.05	0.013	357 (196-1563)/∞ (354-∞)	Equi/Expa/**Equi**
CH	66 (35, 31)	11.58 ± 1.41	7.06 ± 0.82	0.58 ± 0.26	0.77 ± 0.07	0.77 ± 0.06	−0.011	48 (42-55)/∞ (23-∞)	Bott/Equi/**Equi**
TF	24 (13, 11)	9.67 ± 1.40	6.51 ± 0.78	0.25 ± 0.13	0.74 ± 0.06	0.74 ± 0.05	−0.008	490 (63-∞)/∞ (21-∞)	Equi/Expa/**Equi**
NI	183 (91, 92)	13.08 ± 1.76	6.40 ± 0.64	1.25 ± 0.33	0.75 ± 0.04	0.75 ± 0.05	0.002	171 (145-205)/∞ (29-∞)	Bott/Expa/**Equi**
DR	9 (6, 3)	2.25 ± 0.46	2.25 ± 0.46	0.08 ± 0.08	0.22 ± 0.08	0.25 ± 0.09	0.026	—	—

Shown are mean ± SE for allelic richness (*N*_*a*_), the number of private alleles (*N*_*p*_), observed heterozygosity (H_O_), and the unbiased expected heterozygosity (H_e_). The Wright’s fixation index for within-population inbreeding (F_IS_) corresponds to a mean value across all loci. For the effective population size (*N*_*e*_) mean values and 95% confidence intervals are presented.

–: not determined due to low sample size.

*Rarefied allelic richness estimated for the lowest sample size among localities (n = 9).

**Effective population size estimated with the linkage disequilibrium (left) and heterozygosity-excess (right) methods in NeEstimator 2.1.

^Recent demographic changes inferred from the infinite alleles model (IAM; left), stepwise mutation model (SMM; center), and best two-phase mutation model (TPM; right, in bold) using BOTTLENECK 1.2.05. ‘Bott’: recently bottlenecked; ‘Expa’: recent population expansion; ‘Equi’: mutation-drift equilibrium.

### Field procedures and genotyping

We captured 582 adult rayaditos (292 females and 290 males) using mist nets or mechanical traps inside nestboxes. Birds were marked using individual numbered aluminum bands and bled by brachial venipuncture. All birds were released near the capture site immediately after handling. Details on capture methods and field procedures are given in Supplementary Information Appendix 1. The methodology was approved by the Ethics Committee of the Sciences Faculty, Universidad de Chile following guidelines from Biosecurity Manual from CONICYT (version 2008) and Chilean law 20380 about animal protection. Full permission for sampling and animal ethics approval were granted by Servicio Agrícola y Ganadero (SAG; permits Nos. 5193/2005, 6295/2011, 1101/2013, 7542/2015, 5158/2016, 8185/2016, 404/2017, 4209/2017, 2667/2018) and Corporación Nacional Forestal (CONAF), Chile, and Administración de Parques Nacionales (APN; research project No. 1405), Argentina.

We extracted DNA from blood samples and genotyped all individuals at 12 autosomal polymorphic microsatellite loci. Primer sequences and PCR conditions are described in Botero-Delgadillo *et al*.^25^ (see also Supplementary Information Appendix 1). We determined the sex of each individual by amplification of the CHD locus using the primers P2/P8^40^.

### Preliminary analyses

We tested for deviations from Hardy-Weinberg equilibrium (HWE) and estimated the frequency of potential null alleles at each locus in each population. Additionally, we tested for linkage disequilibrium between all pairs of loci in each locality. These analyses were performed in the adegenet^41^ and poppr^42^ packages in the free software R 3.5.2^43^. Only two loci in the CH population and one in MA deviated from HWE (Supplementary Fig. S2). Frequencies of null alleles of ~0.1 were estimated for only one locus in CH and one in MA (Supplementary Table S1). Values for the standardized index of association $\bar{r}\,$_d_ were all <0.1, suggesting low covariation among loci (Supplementary Fig. S3). We thus considered all loci for further analyses.

We assessed standard measures of genetic diversity for each population using the packages poppr and hierfstat^44^ in R. We also estimated contemporary population effective sizes and tested for genetic signals of recent demographic changes based on allele frequency data. Estimation of effective sizes and demographic inference were implemented in the programs NeEstimator 2.1^45^ and BOTTLENECK 1.2.02^46^, respectively. Parameter settings and models are specified in Supplementary Information Appendix 1.

### Assessing isolation by distance (IBD)

We performed a Mantel test^47^ to calculate the relationship between genetic and geographic distances using the adegenet package in R. We tested for IBD by comparing the observed correlation with a randomly generated distribution of simulated correlations under no IBD. For this, we first generated matrices of genetic (Classical Euclidean or Rogers’distance^48^) and geographic (Euclidean) distances, and then calculated Spearman rank correlations for 1000 matrix permutations^49^. We created plots of geographic vs. genetic distances and measured local density of points with a 2-dimensional kernel estimation, to determine whether the data are consistent with a single genetic cline or whether there is evidence for two or more regional clusters^50^.

### Evaluation of population genetic structure

We used five different approaches to evaluate range-wide genetic structure. First, we calculated G-statistics for all pairs of sampled localities in GenAlEx 6.5^51^, estimating both the Nei´s standardized index (*G’*_*ST(Nei)*_)^52^ and the Hedrick’s standardized index corrected for small samples (*G”*_*ST*_)^53^. Second, we conducted a Principal Component Analysis (PCA) as implemented in the adegenet package to assess genetic substructure^54,55^. Third, we used the *snapclust* clustering algorithm^56^, also available in adegenet, to infer the number of genetic clusters present throughout the species’ range. This method performs similarly to standard clustering methods, but rapidly converges to a maximum-likelihood solution by combining a geometric approach and the Expectation-Maximization algorithm^56^. We tested values of K –i.e. the number of genetically distinct clusters– between 1 and 10, and determined the optimal number of groups using the Akaike Information Criterion (AIC). Fourth, we implemented a Discriminant Analysis of Principal Components (DAPC^57^) as a complementary approach, given the potential bias introduced by IBD in the PCA and *snapclust* results^50,58^. The DAPC outperforms standard clustering algorithms when genetic structure is more or less continuous, making it ideal for assessing genetic structure under complex dispersal scenarios such as those represented by the stepping-stone and hierarchical stepping-stone models^57^. This method uses sequential K-means clustering and the Bayesian Information Criterion (BIC) to infer the number of genetic clusters. We selected the number of retained PCs for the DAPC to optimize the *a*-score (optimal number: 21), using the optim.a.score function in adegenet. Last, we performed Hierarchical Analyses of Molecular Variance (AMOVA) using the poppr package to quantify the partitioning of genetic variation at different levels. For this, we entered each genetic cluster identified by *snapclust* as ‘region’ and each sampled locality as ‘population’. We provide further details on these analyses in the Supplementary Information Appendix 1.

### Estimating contemporary gene flow

We estimated the rate and direction of recent gene flow using BayesAss 3.0.4^59^. This program calculates migration rates (hereafter referred to as dispersal rates) over the last few generations and does not assume HWE or mutation-drift equilibrium^59^. We estimated dispersal rates (i) for all sampled populations and (ii) for the genetic clusters identified by *snapclust*. For each analysis, we used default parameters for allelic frequency (*a*), gene flow rate (*m*), and inbreeding (*f*) for a first run. In subsequent runs, we modified delta values –i.e. the maximum amount a parameter can change in each iteration– to ensure that proposed changes in parameters were between 20–40%^59^. Adjusted final delta values are provided in the Supplementary Information Appendix 1. Each analysis was run three additional times with 30 million iterations and different random seeds, a sampling frequency of 2000, and a burn-in of 10%. A visual inspection of the continuous parameters sampled from the Bayesian MCMC in the program Tracer 1.7.1^60^ showed that the chains did converge for both analyses (Supplementary Fig. S4).

### Analyses using a reduced data set

Sample sizes among populations were uneven (see Supplementary Information Appendix 1). To minimize potential biases in our results (e.g. biased estimation of dispersal rates; see Faubet *et al*.^61^), we replicated analyses of genetic structure and dispersal rates using a reduced, relatively even, subset of data. Simulation studies recommend using 40–60 individuals per population for implementing Bayesian analyses^60^. Consequently, the reduced data set consisted of 40 randomly selected individuals from each of the populations with large sample sizes –i.e. FJ, MA, BA, CH, and NI–, and all individuals from the remaining localities. Analyses using the reduced data yielded similar results and are only briefly described in the Results.

## Results

### Genetic diversity and effective population size

Genetic diversity was highest in the center of the species’ breeding range (populations BA and CH), although the southern populations (TF and NI) also showed high diversity (Table 1). Allelic richness and heterozygosity levels were extremely low in the insular population of DR, while values for the northernmost localities were moderately low (Table 1). Genetic diversity estimates were similar when using the reduced data set (Supplementary Information Appendix 4).

Estimates of effective population size were higher in those populations with the highest genetic diversity, except for CH (Table 1). Genetic evidence for recent bottlenecks or population expansions was not conclusive (Table 1). Different mutation models showed that in all sampled populations values of observed heterozygosity did not deviate from expectations under mutation-drift equilibrium (Table 1). However, a distant or weak bottleneck in MA cannot be discarded (Supplementary Table S2).

### Isolation by distance (IBD)

**Figure 2:**
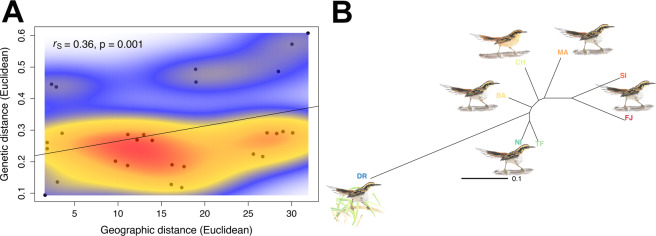
Graphic illustration of isolation by distance (IBD) across the breeding range of thorn-tailed rayadito based on 582 individuals from eight populations. (**A**) Scatterplot of geographic vs. genetic distance and 2-dimensional kernel density estimation to assess local density of points. A gradient from low to high density is represented by a blue-to-red color palette. Included are the estimated correlation coefficient with a simulated p-value based on 1000 permutations to test for IBD. (**B**) A neighbor-joining tree representing genetic distance (Euclidean or Rogers’ distance) between populations. Shown are the currently recognized subspecies included in this study.

The Mantel test performed on the matrices of genetic and geographic distances detected IBD (*r*_S_ = 0.36, p = 0.001; Fig. 2A). The 2-dimensional kernel density estimation showed one main cloud of points, although there were two smaller clusters that appeared slightly separated (Fig. 2A). When removing the DR population –the most distant population in genetic space (Fig. 2B)–, evidence for IBD became weaker (*r*_S_ = 0.33, p = 0.05; Supplementary Fig. S5). However, after subsequently removing the FJ population –the second most differentiated population (Fig. 2B)–, the IBD signal became stronger again (*r*_S_ = 0.64, p = 0.02; Supplementary Fig. S5).

### Range-wide genetic structure

Signatures of genetic structure were detected with all four analytical approaches. Genetic distances ranged from 0.001 (between TF and NI) to 0.45 (between FJ and DR) when using the *G’*_*ST(Nei)*_ index, and from 0.005 to 0.85 with the *G”*_*ST*_ index (Table 2). Regardless of the estimate used, pairwise G-statistics revealed that the population in DR was strongly differentiated from the other populations (Table 2). Rayaditos from FJ were also moderately differentiated from all populations south of SI (Table 2). This was similar to the pattern shown in Fig. 2B.

**Table 2:** G-statistics for each pair of sampled populations across the breeding range of thorn-tailed rayadito.

Locality								
	FJ	SI	MA	BA	CH	TF	NI	DR
FJ	—	0.217	0.385	0.411	0.334	0.398	0.420	0.855
SI	0.067	—	0.242	0.362	0.293	0.342	0.354	0.794
MA	0.112	0.068	—	0.214	0.172	0.226	0.245	0.689
BA	0.112	0.095	0.053	—	0.149	0.037	0.061	0.612
CH	0.092	0.078	0.043	0.034	—	0.203	0.212	0.697
TF	0.115	0.095	0.059	0.009	0.050	—	**0.005**	0.574
NI	0.119	0.097	0.063	0.015	0.051	**0.001**	—	0.592
DR	0.450	0.416	0.346	0.295	0.338	0.287	0.291	—

Values below the diagonal correspond to the Nei’s standardized index (*G’*_*ST(Nei)*_), while values above the diagonal give the Hedrick’s standardized index corrected for small samples (*G”*_*ST*_)_._

Except for the values in bold, all p < 0.001 (based on 1000 permutations). For both values in bold p = 0.28.

**Figure 3:**
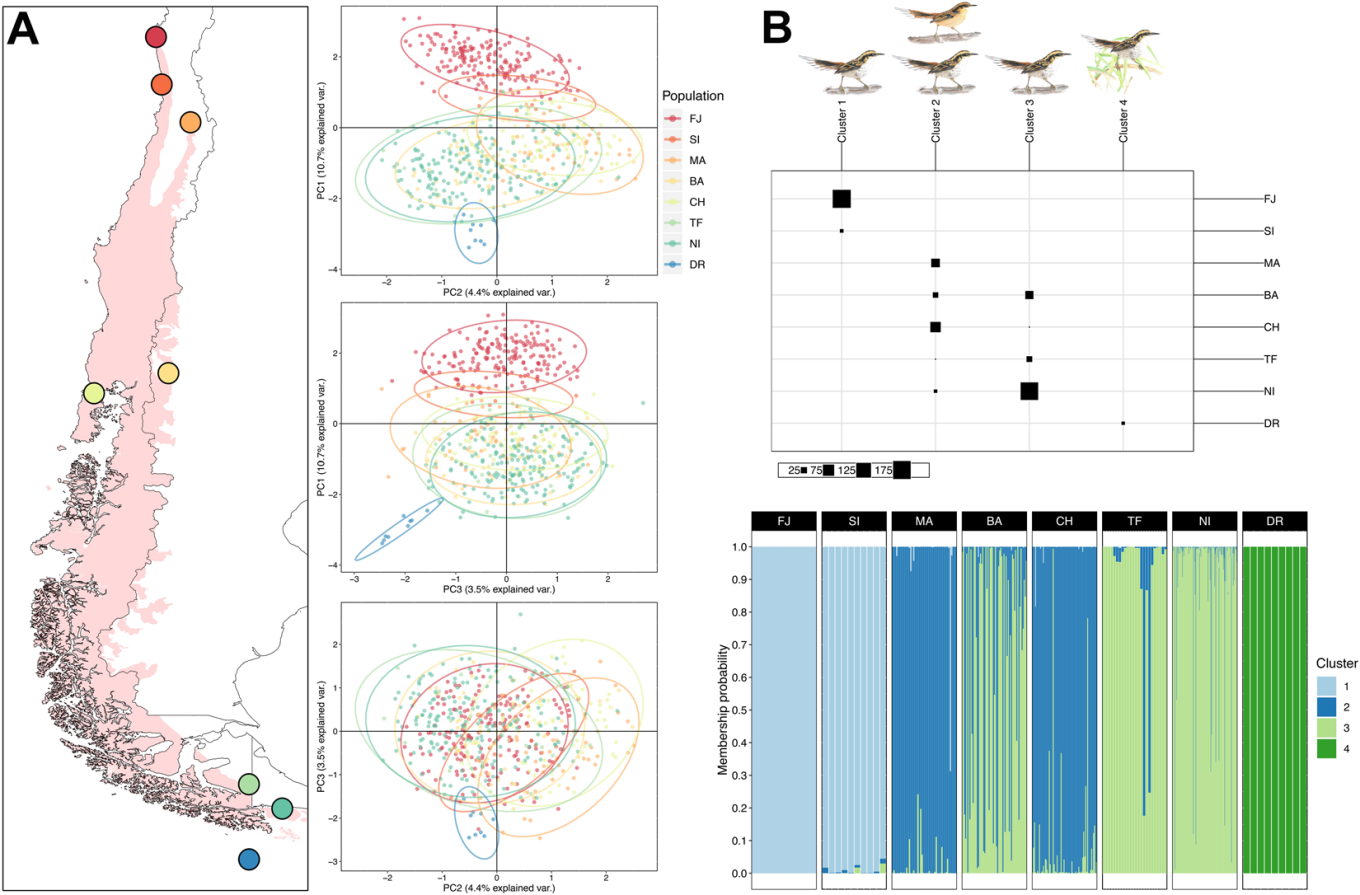
Population genetic structure based on analyses of 582 individuals of thorn-tailed rayadito from eight populations across the breeding range. (**A**) Scatterplots of the first three principal components from a PCA. Each color represents a population with 95% CI shown as ellipses. (**B**) Results from the *snapclust* analysis. Above: population assignment to four genetic clusters identified in the analysis (K4) and representation of currently recognized subspecies in each cluster. Below: assignment of individuals from different populations to clusters shown as different colors. Each vertical line represents an individual. Individuals are grouped according to their population of origin.

In the PCA, we retained 46 dimensions that explained 80% of the total genetic variance. Although variation was more or less continuous, three to four groups could be discerned when plotting the first three components (19% of total variance; Fig. 3A).

According to the *snapclust* method, the most likely number of genetic clusters in our sample ranged from three to seven. Although the lowest AIC value was obtained for K7, values plateaued between K3 and K4, and then slightly increased for K5 (Supplementary Fig. S6). We thus selected K4 as the optimal number, although we also used K3 for further analyses. Group assignments with K4 resulted in the following arrangement: (1) a northern cluster, consisting of FJ and SI; (2) a north-central cluster, comprised by MA and CH; (3) a south-central cluster, formed by BA, TF, and NI; and (4) the DR population (Fig. 3B). Individuals forming these clusters had high membership coefficients, although birds from BA showed ‘mixed’ origin (~33% of the sample was assigned to the north-central cluster; Fig. 3B). Group assignments with K3 resulted in the DR and the south-central group being merged into one southern cluster, whilst the other groups were configured similarly (Supplementary Fig. S7).

**Figure 4:**
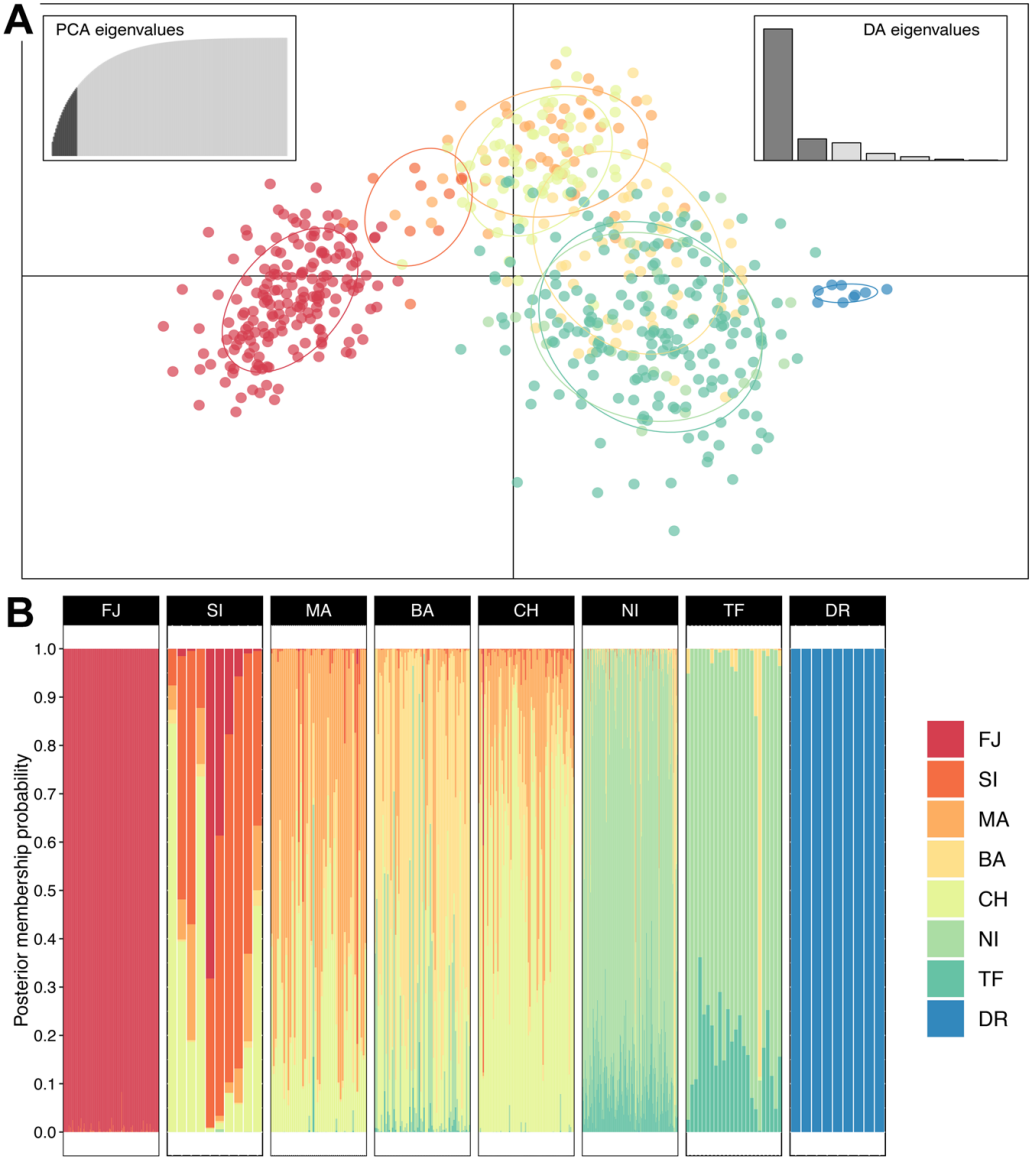
Population genetic structure based on a Discriminant Analysis of Principal Components (DAPC) based on 582 individuals of thorn-tailed rayadito from eight populations. (**A**) Scatterplot of the first two discriminant functions, based on 21 retained principal components. Each color represents a population with 95% CI shown as ellipses. Eigenvalues for principal components and discriminant functions are depicted. (**B**) Individual membership probabilities to each population based on seven retained discriminant functions. Here, populations were used as prior clusters. Each vertical line represents an individual.

Comparable with the *snapclust* method, the K-means clustering from the DAPC indicated that the most probable number of clusters ranged from three to six. The lowest BIC value was obtained for K10, but values plateaued after K6 (Δ_BIC K6–K7_ = 5.14; Supplementary Fig. S6). For the DAPC, 21 principal components (58% of total variance) and seven discriminant functions were retained. The pattern of genetic structure revealed by the DAPC was very similar to the one observed with the PCA (Fig. 4A). Close inspection of the membership probabilities to each locality showed that the genetic composition of populations (Fig. 4B) was analogous to the pattern of population clustering presented in Fig. 3B. However, SI appeared more differentiated from the FJ and MA populations (Fig. 4).

Hierarchical AMOVAs showed conclusive evidence for a genetic structure between clusters and between populations within clusters (Table 3). These analyses showed that 8–9% of the total genetic variance was explained by partitioning samples into either four –i.e. north, north-central, south-central, and DR– or three –i.e. north, north-central, south– regional genetic clusters (Table 3). Variance between the sampled populations within the clusters corresponded to an additional 2–4% (Table 3).

**Table 3:** Results of hierarchical AMOVAs showing genetic variation partitioning across the breeding range of thorn-tailed rayadito.

Source of variation*	df	SS_T_	MS_T_	% Variation	p-value^
	Four genetic clusters (K4)				
Between clusters	3	725.09	241.69	8.1	0.01
Between populations within clusters	4	126.92	31.73	2.4	0.01
Between samples within populations	574	5025.90	8.75	0.8	0.17
Within samples	582	5007.00	8.60	88.8	
Total	1163	10884.92	9.35	100	
	Three genetic clusters (K3)				
Between clusters	2	595.26	297.63	8.5	0.009
Between populations within clusters	5	256.74	51.34	3.8	0.01
Between samples within populations	574	5025.90	8.75	0.8	0.041
Within samples	582	5007.00	8.60	86.9	
Total	1163	10884.92	9.35	100	

Genetic clusters were defined using the *snapclust* method (see text).

^*^Genetic clusters were entered as ‘region’. Fixation indices with K4: F_SC_ = 0.0088, F_ST_ = 0.026, F_CT_ = 0.081. Fixation indices with K3: F_SC_ = 0.0088, F_ST_ = 0.042, F_CT_ = 0.085.

^p-values derived from Monte-Carlo tests based on 1000 permutations to test whether genetic variance explained by partitioning data into clusters/sexes and populations/clusters was greater than expected from randomly generated values.

We found similar results for all analyses of genetic structure with the reduced data set (Supplementary Information Appendix 4). The *snapclust* method identified 4–5 genetic clusters. The four clusters are the same as those shown in Fig. 3B. The additional cluster in the K5 model resulted from the separation of the north-central group, such that the MA and CH populations comprised their own cluster.

### Contemporary gene flow

**Figure 5:**
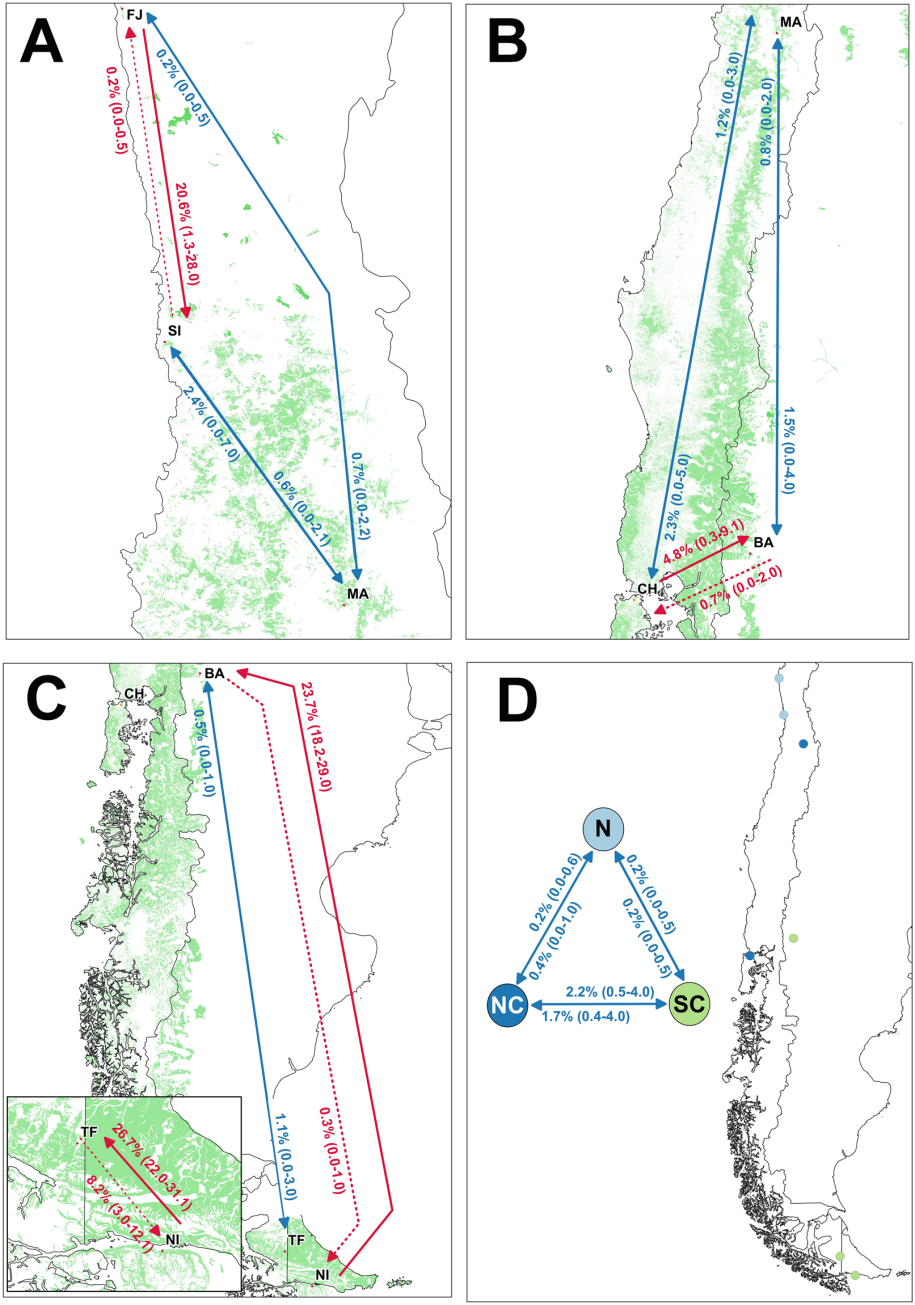
Illustration of levels of contemporary gene flow among populations and genetic clusters across the breeding range of thorn-tailed rayadito. Reported are mean (with 95% confidence intervals) dispersal rates –i.e. the proportion of immigrants in a population–, estimated using BayesAss based on 573 individuals. Red arrows indicate biases in directionality of gene flow, with the dashed line indicating the lower dispersal rate between a pair of populations/clusters. Blue arrows represent relatively symmetric gene flow between populations/clusters. Panels (**A**–**C**) illustrate gene flow among seven ‘continental’ populations of rayadito (K7). Panel (**D**) shows gene flow among three identified ‘continental’ genetic clusters (K3).

Estimates of dispersal rates indicated that there was no recent gene flow between DR and the other sampled localities (*m* = 0 in all cases). We therefore show results from analyses excluding this population. A first analysis estimates gene flow between the remaining populations (hereafter ‘continental’ populations; K7), while a second analysis was based on the remaining genetic clusters –i.e. north, north-central, and south-central clusters (hereafter ‘continental’ genetic clusters; K3). Reported mean dispersal rates were obtained from the MCMC run with the smallest value of -2 log Pr(*X*/K) and the largest effective sample size (Fig. 5; Supplementary Tables S3, S4).

The first analysis (K7) shows asymmetric gene flow between geographically isolated populations and their neighboring localities –i.e. between FJ and SI, CH and BA, NI and TF, and NI and BA (Fig. 5). As expected, dispersal rates were lower towards the isolated populations than from them. The second analysis (K3) indicates moderately low gene flow between the north-central and south-central clusters, whilst the northern cluster appears more isolated (Fig. 5D). Among all cases with mean dispersal rates > 0.01 (20 out of 42), the estimated proportion of dispersers between localities ranged from 1.1% (MA to TF) to 26.7% (NI to TF), and between clusters from 1.7% (south-central to north-central) to 2.2% (north-central to south-central).

Although estimates of gene flow were similar for the reduced data set, values were in general slightly higher (Supplementary Information Appendix 4). However, changes in dispersal rates rarely exceeded 0.01 (4 out of 42 cases), and patterns of demographic interactions among populations/clusters remained as shown in Fig. 3 (Supplementary Information Appendix 4). Only the estimate of gene flow from BA to CH noticeably increased (17.4% versus 0.6% in the complete data set).

## Discussion

Results from a variety of methods showed that populations from the edge of the breeding range of the thorn-tailed rayadito were more differentiated, with evidence for the existence of three to five genetic clusters. Analyses based on both the complete and the reduced data sets identified four genetic clusters that followed a clear geographic pattern: a northern cluster (FJ and SI), a cluster on the west side of the Andes (MA and CH), a cluster east of the Andes extending south to Patagonia (BA, TF, NI), and the rayaditos from the Diego Ramírez Archipelago. We also found genetic evidence for gene flow across the species’ distribution. Below we discuss these findings starting with the demographic implications of the observed patterns of genetic diversity.

Patterns of allelic richness and heterozygosity are informative about the role that stochastic processes may have played in the recent dynamics of populations. During founder effects or population bottlenecks, rare alleles are readily lost^62^. The low number of private alleles and the moderately high levels of heterozygosity in FJ and SI (Table 1) suggest a limited effect of genetic drift on these isolated populations. Our results indicate that the effective population size in FJ is comparable to that of more diverse populations, suggesting that this population did not suffer dramatic changes in the recent past. This is not unexpected given that the isolation of forest relicts in this region has been a gradual, long-term process^36,37^. In the case of the small forest of SI, immigration from other populations (e.g. FJ or MA) might keep up genetic variation, possibly counterbalancing the potential effect of genetic drift due to a reduced population size. Although we had no genetic evidence of a reduced population in SI, capture-mark-recapture data show that the breeding population is small (53–106 individuals; Botero-Delgadillo, unpublished). The high genetic diversity observed in the southern populations confirms findings from a previous study^27^, and supports the hypothesis of an austral paleorefugium in Tierra del Fuego from which rayaditos colonized different localities as the ice retreated and forests expanded during the Holocene. The severely reduced genetic diversity found in DR could be a combined effect of a recent founder event, genetic drift, inbreeding, and strong selection in the extreme environment of this isolated archipelago^63^.

Genetic structure was pronounced at the between-region level, but not between populations within regions, at least along the more continuous part of the species’ range –i.e. from MA down to NI. The relatively low genetic structure revealed by the AMOVAs (<10% of the total variance explained) likely reflects the interplay of moderate genetic connectivity and ongoing gene flow that might be limited by strong landscape resistance, particularly towards the range margins. A previous study using mitochondrial sequence data (Cytb) and inter-simple-sequence-repeats (ISSR) from 8 populations (including FJ, CH and NI)^27^ also reported low levels of among-population genetic variance (~11% of variance explained in an AMOVA).

Results from the AMOVAs, along with the observed correlation between latitude and the first principal component of the PCA, could be indicative of continuous population differentiation in our study system. This might have biased some of our results, because assignment methods may erroneously detect the presence of discrete clusters in the presence of isolation by distance (IBD)^50,58,64^. This can be particularly problematic when spatial sampling is sparse^64,65^, as in this study. However, we suggest that our data support a more complex scenario in which range-wide genetic structure is not strictly continuous, but follows a hierarchical clustered pattern^66,67^. For instance, visual inspection of IBD suggests more than one cloud of points (Fig. 2A). More importantly, the evidence for IBD became weaker after removing the genetically most distant population (i.e. DR). This is in line with the observation that classical IBD is not only caused by the presence of a continuous cline of genetic variation, as distant and differentiated populations can also produce such a pattern^4,5^. Interestingly, the strongest correlation between genetic and geographic distances was obtained after further excluding FJ from the analysis. This suggests that clinal genetic variation might be highest for ‘continental’ populations where forest habitat is more continuously distributed (south of MA; Supplementary Fig. S1). We acknowledge that the main shortcoming of our study is the fact that we sampled at a limited number of locations across the species’ distribution, and that a more continuous sampling is necessary to test the hypothesis of a range-wide hierarchical genetic structure.

Although the DAPC is not immune to the potential biases related to spatially heterogeneous sampling, it provides robust results under different scenarios, including a hierarchical stepping-stone model^57^. Results from this approach were concordant with the possibility of clinal variation of genetic diversity in certain parts of the distribution of thorn-tailed rayadito, but also with the presence of distinct clusters. Environmental and physical barriers to dispersal may be responsible for this, but this needs to be further investigated.

The range-wide patterns of genetic diversity and population genetic structuring in rayaditos are consistent with the central-marginal model, which argues that geographically peripheral populations exhibit reduced diversity and higher genetic differentiation than core populations^68^. The model also predicts that populations are more sparsely distributed towards the range edge^68,69^. Although this appears to be the case in the northern range margin, it is clearly due to the reduced availability of suitable habitat, whereas populations in the south exhibited high diversity and connectivity, as available habitat is widespread (Supplementary Fig. S1).

Despite the fact that rayaditos are considered forest specialists whose local movements are restricted by landscape structure^24,25,39^, colonization of oceanic islands has been interpreted as an indication of high dispersal capacity^23^. The study by González and Wink^27^ and our results support this view, providing evidence for range-wide gene flow in this species. Although breeding seems mostly restricted to old-growth and secondary native forests^29^, rayaditos use industrial plantations and early successional vegetation as foraging habitats^30^. The use of these habitats enhances connectivity between populations breeding in native forest remnants^70^. Remarkably, there are other reported cases of high dispersal capability in forest-dwelling birds that are reluctant to cross open habitats at the local scale^19^. It remains to be determined whether these movements result from few long-distance dispersal bouts carried out in a short period or whether they are the sum of sequential movements of vagrant juvenile individuals in search of suitable and available breeding habitat (see Menger *et al*.^21^).

Estimates of contemporary gene flow supported the genetic structure analyses and showed that there was no gene flow from or towards the population in DR. Bayesian analyses indicated that ‘continental’ populations and clusters from MA southwards had non-independent demographic dynamics –i.e. the 95% CI around the mean dispersal rates did not include zero^61^. These analyses also showed that FJ and SI could be considered demographically independent from southerly populations. Care must be taken when interpreting these estimates, because these only represent the proportion of immigrants in a given population or cluster, and not the proportion of emigrants^59^. For instance, birds from FJ comprised a large proportion within the small sample from SI, but this does not reflect the fraction of actual emigration from FJ, which could be very low.

Asymmetric gene flow was more notable in the northern and southern part of the species’ breeding range. In the north, this may reflect source-sink population dynamics between FJ and SI. Although this requires further study, we hypothesize that demographic differences rather than landscape configuration are the main cause underlying this pattern. Because the landscape is highly fragmented and rayaditos moving between FJ and SI are likely to encounter the same resistance to dispersal, more individuals will move from the larger into the smaller population than vice versa^68^. The larger influx of rayaditos from CH and NI to the continent as compared to the island is harder to explain, but was also found by González and Wink^27^. Limited breeding space on these islands could force birds to move to the nearby continent.

It is worth noting that the analyses based on the reduced data set yielded similar results to those with the complete data set. It is well known that individual assignments and immigration rates are more accurate when using genetic clusters previously identified instead of sampled populations, as clusters comprise more clear-cut groups^59,61^. However, both population- and cluster-based analyses indicated gene flow between populations from the eastern and western sides of the Andes and down to Patagonia.

Despite the limitations of our sampling scheme, the correspondence between different methodological approaches suggests that our results are robust. In addition to the implications regarding the distribution of genetic variance in our study species, there are four relevant aspects regarding the conservation of local populations of rayadito that can be drawn from this study.

1. Based on our characterization of 12 putatively neutral genetic markers, we found that populations at the range margins were genetically differentiated from more central populations. Our results confirm the long-held assumption that the FJ population is relatively isolated from other populations of rayadito (see Quirici *et al*.^71^). Nevertheless, the population does not seem affected by strong genetic drift in the recent past, but future studies should determine the level of inbreeding depression. The DR population is clearly genetically depauperate and different from other populations. Thus, conservation measures should be given high priority given the population’s potential value as a source for future speciation events (Rozzi *et al*., unpublished). Whether this apparent genetic singularity also involves unique adaptive genetic variation requires further investigation.
2. The small population size in SI and DR imply an increased risk of local extinction due to stochastic events. Loss of genetic diversity is also a risk in the long term.
3. The southern populations are important because they maintain gene flow with central populations, and because they harbor high levels of genetic diversity. The latter could make them more resilient to perturbations and stochastic processes.
4. Human activity has increased the degree of habitat fragmentation in central Chile, which may compromise the long-term persistence of rayaditos in MA and the entire region^24,39^. A reduced effective population size and the possibility of a distant bottleneck suggest that this population may be highly inbred. Future studies can investigate the effects of inbreeding and the presence of behavioral mechanisms to avoid it.

To conclude, we summarize the main implications of our study for the current taxonomic treatment of the sampled populations and subspecies. Note that the case of the DR rayadito will be treated in detail elsewhere (Rozzi *et al*., unpublished).

1. The clear genetic structuring and restricted gene flow in the northern part of the species’ breeding range suggest that FJ and SI should be considered as a demographically independent unit.
2. All subspecies of thorn-tailed rayadito are currently described based on morphology and plumage coloration. Although González and Wink^27^ found evidence of genetic differentiation of the subspecies *A. s. bullocki* (not sampled here) relative to other subspecies, they observed weak differentiation between *A. s. fulva* and the nominate subspecies. In agreement with this, our results indicate gene flow between *A. s. fulva* and *A. s. spinicauda*. It has been suggested that the ochraceous underparts –typical of *fulva* but also present in *bullocki* to a lesser extent– may have appeared independently in insular populations near mainland Chile in response to the more humid conditions found on these islands –following Gloger’s Rule^23^. A phylogeographic study is needed to assess the validity of *A. s. fulva* as a subspecies.
3. González and Wink^27^ suggested the potential presence of a non-described Austral subspecies. Although we here describe neutral genetic variation only, our data suggest that populations on the east side of the Andes (e.g. BA) and those further south down to Patagonia may constitute one highly diverse genetic cluster within which moderate levels of gene flow are maintained.

## Supplementary information

Supplementary Information.
