# Supplementary material for: Range-wide genetic structure in the thorn-tailed rayadito suggests limited gene flow towards peripheral populations
Source: Sci Rep. 2020 Jun 10;10:9409. doi: 10.1038/s41598-020-66450-7 (PMC7287099; doi:10.1038/s41598-020-66450-7)
Supplement: Supplementary file 1 — Supplementary Information. [file 41598_2020_66450_MOESM1_ESM.docx]

**Supplementary information**

Range-wide genetic structure in the thorn-tailed rayadito suggests limited gene flow towards peripheral populations

Esteban Botero-Delgadillo^1,2,3,*^, Verónica Quirici^4,5^, Yanina Poblete^1,6^, Matías Acevedo^7^, Élfego Cuevas^8^, Camila Bravo^1^, Margherita Cragnolini^2^, Ricardo Rozzi^9,10^, Elie Poulin^1^, Jakob C. Mueller^2^, Bart Kempenaers^2^, & Rodrigo A. Vásquez^1^

^1^Instituto de Ecología y Biodiversidad, Departamento de Ciencias Ecológicas, Facultad de Ciencias, Universidad de Chile, Santiago, Chile

^2^Department of Behavioural Ecology and Evolutionary Genetics, Max Plank Institute for Ornithology, Seewiesen, Germany

^3^SELVA: Research for conservation in the Neotropics, Bogotá, Colombia

^4^Departamento de Ecología y Biodiversidad, Facultad de Ecología y Recursos Naturales, Universidad Andrés Bello, Santiago, Chile

^5^Centro de investigación para la sustentabilidad, Universidad Andrés Bello, Santiago, Chile

^6^Instituto de Ciencias Naturales, Universidad de las Américas, Santiago, Chile

^7^Programa de Magister en Áreas Silvestres y Conservación de la Naturaleza, Facultad de Ciencias Forestales y Conservación de la Naturaleza, Universidad de Chile, Santiago, Chile

^8^Doctorado en Medicina de la Conservación, Facultad de Ecología y Recursos Naturales, Universidad Andrés Bello, Santiago, Chile

^9^Programa de Conservación Biocultural Sub-Antártica, Parque Etnobotánico Omora, Universidad de Magallanes & Instituto de Ecología y Biodiversidad, Santiago, Chile

^10^Sub-Antarctic Biocultural Conservation Program, Department of Philosophy and Religion & Department of Biological Sciences, University of North Texas, Denton, TX, USA

*Corresponding author: Esteban Botero-Delgadillo, eboterod@gmail.com

# **Table of contents**

[Appendix 1: Supplementary methods 2](#_Toc24141389)

[Appendix 2: Supplementary figures 5](#_Toc24141394)

[Appendix 3: Supplementary tables 13](#_Toc24141401)

[Appendix 4: Analyses with a reduced data set 18](#_Toc24141406)

[References: 25](#_Toc24141411)

# **Appendix 1: Supplementary methods**

# **1. Field procedures and genotyping**

We sampled eight localities throughout the breeding range of thorn-tailed rayadito, capturing and bleeding 582 adult individuals. From north to south, the eight localities were: Fray Jorge National Park (FJ; 30º38’ S, 71º40’ W); Cerro Santa Inés (SI; 32º10’ S, 71º30’ W); Cerro Manquehue (MA; 33º21’ S, 70º34’ W); Bariloche (BA; 41º15’ S, 71º16’W); Chiloé Island (CH; 41º52’ S, 73º39’ W); Tierra del Fuego (TF; 54º10’S, 68º42’ W); Navarino Island (NI; 55º4’ S, 67º40’ W); and Gonzalo Island in the Diego Ramírez Archipelago (DR; 56º32’ S, 68º42’ W). Populations from FJ, MA, CH, and NI have been studied during 2007–2018 as part of a long-term research project on the breeding biology of this species (see Botero-Delgadillo *et al*. 2017a, 2017b, 2019, Espíndola-Hernández *et al*. 2017, Ippi *et al*. 2017, Moreno *et al*. 2005, 2007, Quirici *et al*. 2014, 2016). In these study sites, nestboxes have been installed and successfully occupied by rayaditos –occupation rates vary from 0.10 to 0.18 depending on the population. Capture of adult birds in these localities has been carried out during the breeding season by installing a mechanical trap inside occupied nestboxes (Botero-Delgadillo *et al*. 2017a, Quirici *et al*. 2014). As FJ and NI were yearly monitored for at least eight consecutive years, sample sizes from these populations were larger than in MA and CH (see Table 1 in main text). The populations in the remainder localities have not been monitored, thus we used mist netting to capture adult individuals. Due to logistical reasons, these sites were visited only once between 2016 and 2018 –excepting BA–, hence the relatively limited sample sizes (see Table 1 in main text).

Captured birds were marked with a uniquely numbered aluminium band and subsequently bled by brachial venipuncture using a sterile needle. Although nestlings were bled for other purposes, we did not include those individuals in our study in order to reduce the possibility of similar genotypes from siblings being incorporated in the analysis. Blood samples were ~15 µl and were stored on filter paper (FTA Classic Cards, Whatman, Buckinghamshire, UK). DNA was extracted from blood samples using a QIAmp^®^ DNA Micro Kit (QIAGEN^®^ #56304). Rayaditos were genotyped at 12 autosomal polymorphic microsatellite loci, using 7 species-specific markers (see Yáñez *et al*. 2015), and 5 cross-species amplifying markers (see details in Botero-Delgadillo *et al*. 2017a). Although Yáñez *et al*. (2015) originally designed eight markers for rayaditos, we did not include marker As1 as it has shown deviations from Hardy-Weinberg Equilibrium (HWE) in different populations and frequencies of null alleles >0.15 (see Yáñez *et al*. 2015, Botero-Delgadillo *et al*. 2017a). Information on primer sequences, primer concentrations and cycling conditions for multiplex PCR are given in Botero-Delgadillo *et al*. (2017a). All genotypes were double-read independently by two people, and additional PCRs were run for further verification whenever inconsistencies were found (7% of all individuals).

All birds were released near the capture site –or near the breeding nestbox– after marking and handling. Birds were captured and marked under the authority of Servicio Agrícola y Ganadero (SAG; permits Nos. 5193/2005, 6295/2011, 1101/2013, 7542/2015, 5158/2016, 8185/2016, 404/2017, 4209/2017, 2667/2018) and Corporación Nacional Forestal (CONAF), Chile, and Administración de Parques Nacionales (APN; research project No. 1405), Argentina. All field procedures were carried with the supervision of the Ethics Committee of the Sciences Faculty, Universidad de Chile, and Administración de Parques Nacionales, Argentina.

# **2. Preliminary analyses**

Preliminary analyses and tests for neutrality were carried out in the packages adegenet (Jombart 2008), hierfstat (Goudet and Jombart 2015), and poppr (Kamvar *et al*. 2014) in the free software R 3.5.2 (R Core Team 2018). We first tested for deviations from HWE and estimated the frequency of null alleles for each locus in each population. After correcting p-values for false discovery rate (FDR; Benjamini and Hochberg 1995), only two loci in the CH population –loci As25-10 and Tgu05D– and one in MA –locus As07– showed deviations from HWE (Supplementary Fig. S2). Frequencies of null alleles of ~0.1 were estimated for loci Tgu05D in CH and As07 in MA (Supplementary Table 1). Since removal of markers Tgu05D and As07 did not alter our results, all analyses described below were based on information from all loci.

We tested for linkage disequilibrium between all pairs of loci in each locality. Although in four populations the correlation among loci were statistically conclusive, values of the standardized index of association $\overline{r}$_d_ were rather low –the highest being 0.06– (see Supplementary Fig. S3). Overall, values for $\overline{r}$_d_ were always <0.1 (Supplementary Fig. S3), suggesting that covariation among genotyped loci was low.

We characterized the genetic diversity of all populations by estimating allelic richness (absolute and rarefied values), number of private alleles, observed and expected heterozygosity, and the Wright’s fixation index for within-population inbreeding (F_IS_).

For calculating contemporary population effective sizes (*N_e_*), we used the linkage disequilibrium and heterozygosity-excess methods in NeEstimator 2.1 (Do *et al*. 2014). Models included all genotyped loci and assumed random mating. The lowest allele frequency used was 0.02, as the number of independent comparisons markedly increased relative to higher values –i.e. 0.05– and reached a plateau. Reported values correspond to the estimated *N_e_* and the parametric 95% confidence interval around *N_e_* (see Table 1 in main text).

Analyses for detecting genetic signals of recent demographic changes were implemented in BOTTLENECK 1.2.05 (Cornuet and Luikart 1996). Values of expected heterozygosity under mutation-drift equilibrium (H_eq_) were calculated using the infinite alleles model (IAM), the stepwise mutation model (SMM), and the two-phase mutation model (TPM) with varying rates of stepwise mutations –0.3, 0.5, 0.7, and 0.9. A sign test was used to test whether the number of observed loci with heterozygosity excess was different from the expected value under mutation-drift equilibrium (Cornuet and Luikart 1996, Luikart and Cornuet 1998). Given that results under the IAM frequently point at heterozygosity excess –a sign of a recently bottlenecked population–, while under the SMM tend to suggest heterozygosity deficiency –a sign of recent population expansion–, we followed the conservative approach suggested by Luikart and Cornuet (1998) to safely reject the null hypothesis of no excess/deficiency. According to these authors, consistent results should be obtained under both the IAM and SMM models in order to reliably reject the null hypothesis. For instance, both models should suggest a heterozygosity excess/deficiency to safely conclude that a recent bottleneck/expansion took place in a given population. Additionally, because microsatellite markers are suspected to follow a mutation model that is intermediate between the IAM and the SMM (Shriver *et al*. 1993, Di Rienzo *et al*. 1994), we used TPM models with different SMM rates (see above) to determine the best model for each population. A TPM model was selected as the best model whenever the observed heterozygosity values for all loci –or for the highest number observed– did not differ from H_eq_ values expected under the specified parameters (Cornuet and Luikart 1996; see Supplementary Table 2). Recent demographic changes in each population were reported as either ‘bottleneck’, ‘expansion’, or ‘in equilibrium’, depending on results obtained from the IAM, SMM and best TPM models (see Table 1 in main text).

Given that sample sizes should ideally be >20 individuals to achieve reasonable statistical power (see e.g. Luikart and Cornuet 1998), estimates of *N_e_* and recent demographic changes were not inferred for the SI and DR populations. This is also acknowledged in the main text.

# **3. Evaluation of population genetic structure**

Pairwise G-Statistics were calculated in GenAlEx 6.5 (Peakall and Smouse 2012), implementing the Nei´s standardized index (*G’_ST(Nei)_*; Nei 1987) and the Hedrick’s standardized index corrected for small samples (*G’’_ST_*; Hedrick 2005). Nei’s estimator accounts for sampling bias and is robust to variations in sample size, but it can underestimate the value of population structure (Meirmans and Hedrick 2011). Hedrick’s index, on the other hand, is very robust to different sample sizes, but tends to overestimate population structure (Meirmans and Hedrick 2011). As it is likely that the ‘real’ estimate of population structure lies between both estimates, we reported values for both indexes (Meirmans and Hedrick 2011).

We used a PCA as implemented in the adegenet package to explore genetic diversity, and to assess genetic substructure (Patterson *et al*. 2006, Putman and Carbone 2014). We also performed a Principal Coordinate Analysis (PCoA) as an exploratory approach, but given that results were fairly similar to the PCA, only the latter is showed.

Genetic structure was further evaluated by using the *snapclust* clustering algorithm (Beugin *et al*. 2018), which is available in the adegenet package in R. This method relies on the assumption of HWE to compute the likelihood of a given cluster solution, rapidly converging to maximum likelihood estimates of clusters by combining geometric approaches and the Expectation-Maximization algorithm (EM; Beugin *et al*. 2018). We tested values of K –i.e. number of clusters– between 1 and 10 using the ‘snapclust.choose.k’ function, and the optimal number of groups was determined by means of the Akaike Information Criterion (AIC) (see Beugin *et al*. 2018). Individual assignment and calculation of membership probabilities were carried using the ‘snapclust’ function.

Additionally, we used a Discriminant Analysis of Principal Components (DAPC; Jombart *et al.* 2010) to infer the number of genetic clusters throughout the species’ distribution. Given the potential bias introduced by isolation by distance (IBD) in our data, this analysis was used to give further support to results from the PCA and *snapclust*. The DAPC was also implemented in adegenet.

To quantify the partitioning of genetic variation at different levels, we performed Hierarchical Analyses of Molecular Variance (AMOVA) in the poppr package. For these analyses, groups were defined based on the number of clusters identified by *snapclust*. As K3 and K4 were the optimal number of clusters present in our dataset (see *Results* in main text), we fitted two separate AMOVAs. In each analysis, we entered the identified clusters as ‘regions’ and the sampled localities as ‘populations’. We conducted Monte-Carlo tests based on 100 permutations to test whether genetic variance explained by partitioning data into clusters and populations was greater than expected from randomly generated values.

# **4. Estimating contemporary gene flow**

We used BayesAss 3.0.4 (Wilson and Rannala 2003) for calculating rates and direction of recent gene flow between the sampled populations and between the genetic clusters identified by *snapclust*. Previous analyses suggested that rayaditos from the DR population constitute a distinctive genetic cluster (see *Results* in main text), and estimations of dispersal rates evidenced no gene flow between DR and the other sampled localities (*m* = 0 in all cases). Therefore, we reported results from two separate analyses that did not include rayaditos from DR. The first analysis estimated dispersal rates between the ‘continental’ populations –i.e. FJ, SI, MA, BA, CH, TF, NI– (K7), while the second was performed on the three ‘continental’ genetic clusters previously identified (K3) (see *Results* in main text).

In each analysis, a first run was conducted using default parameters for allelic frequency (*a*), gene flow rate (*m*), and inbreeding (*f*). Delta values were modified in subsequent runs to ensure that proposed changes between chains at the end of the run were 20–40% (Wilson and Rannala 2003). Mixing parameters for subsequent runs in the K7 analysis were: ∆*a* = 0.5, ∆*m* = 0.8, ∆*f* = 0.9. For the K3 analysis, delta values were: ∆*a* = 0.2, ∆*m* = 0.7, ∆*f* = 0.8. We ran each analysis three additional times with different random seeds. Results presented were obtained from the MCMC run with the smallest value of -2 log Pr(*X*/K) and the largest effective sample size (Faubet *et al*. 2007). Diagnostics of the MCMC output and convergence were analysed in the software Tracer 1.7.1 (Rambaut *et al*. 2018).

As simulation studies have shown that estimates of migration –or dispersal– rates are less biased for the mean values than for the mode when K > 2 (Faubet *et al*. 2007), we reported mean values and the 95% CI around the mean.

# **Appendix 2: Supplementary figures**

# **Contents:**

Supplementary Figure S16

Supplementary Figure S27

Supplementary Figure S38

Supplementary Figure S49

Supplementary Figure S510

Supplementary Figure S611

Supplementary Figure S712


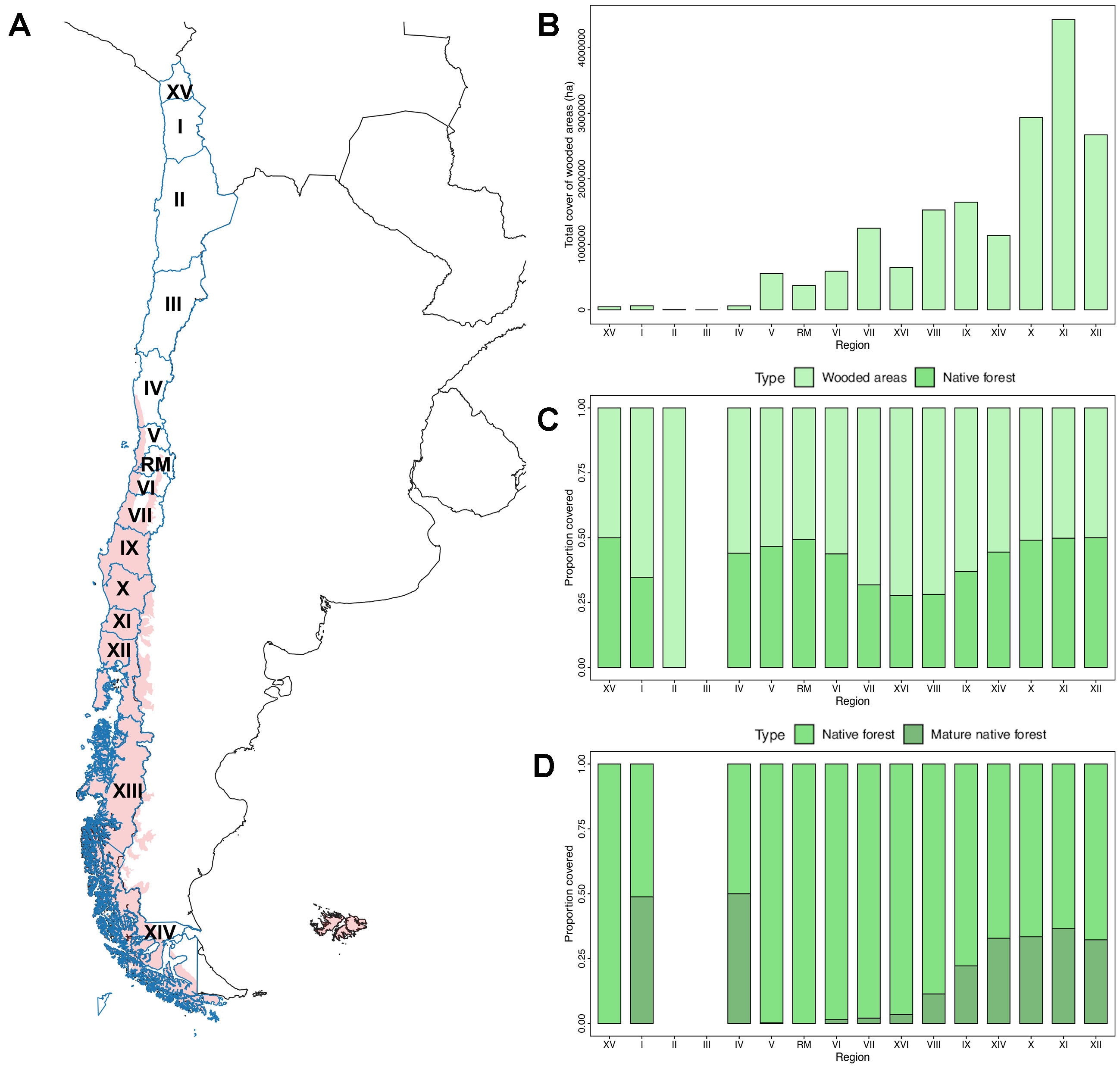


# **Supplementary Figure S1**

Extension of forest habitats across Chile. (**A**) Regional map of Chile and breeding range of thorn-tailed rayadito (light red). Regions in Chile are denoted with roman numbers (except for the metropolitan region, RM). (**B**) Total extension of wooded habitats in 16 regions throughout Chile. (**C**) Area covered by native forests relative to the extension of wooded habitats. (**D**) Area covered by mature forests relative to the extension of native forests.


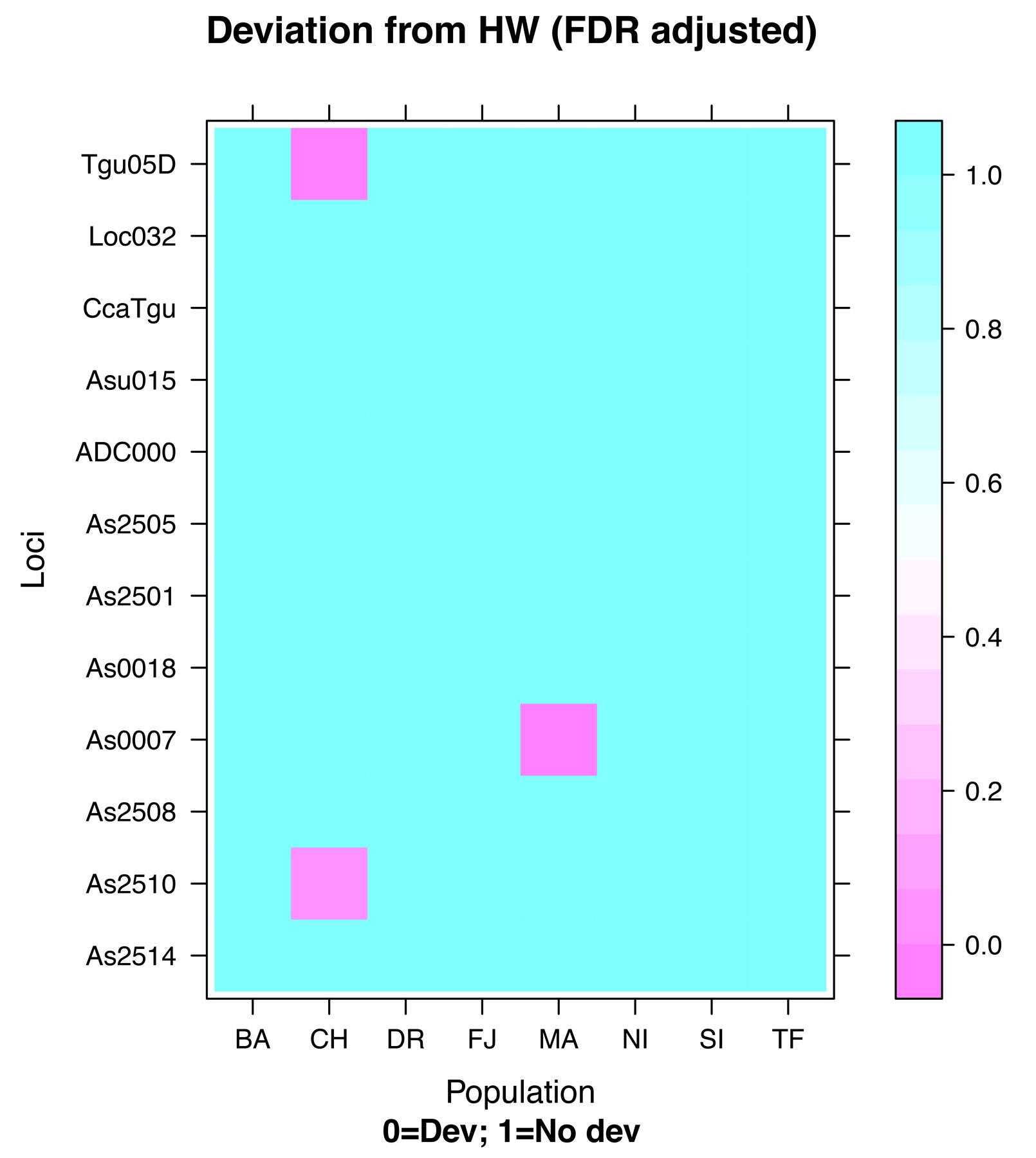


# **Supplementary Figure S2**

Deviations from Hardy-Weinberg Equilibrium (HWE) in 12 polymorphic microsatellite loci sampled in eight populations of thorn-tailed rayadito. FJ: Fray Jorge National Park; SI: Cerro Santa Inés; MA: Cerro Manquehue; BA: Bariloche; CH: Chiloé Island; TF: Tierra del Fuego; NI: Navarino Island; DR: Diego Ramirez Archipelago. Shown are the probabilities that genotype frequencies in each locus differed from expected values under HWE (0 = the locus deviates from HWE; 1 = no deviation). The p-values shown were corrected for false discovery rate (FDR; Benjamini and Hochberg 1995).


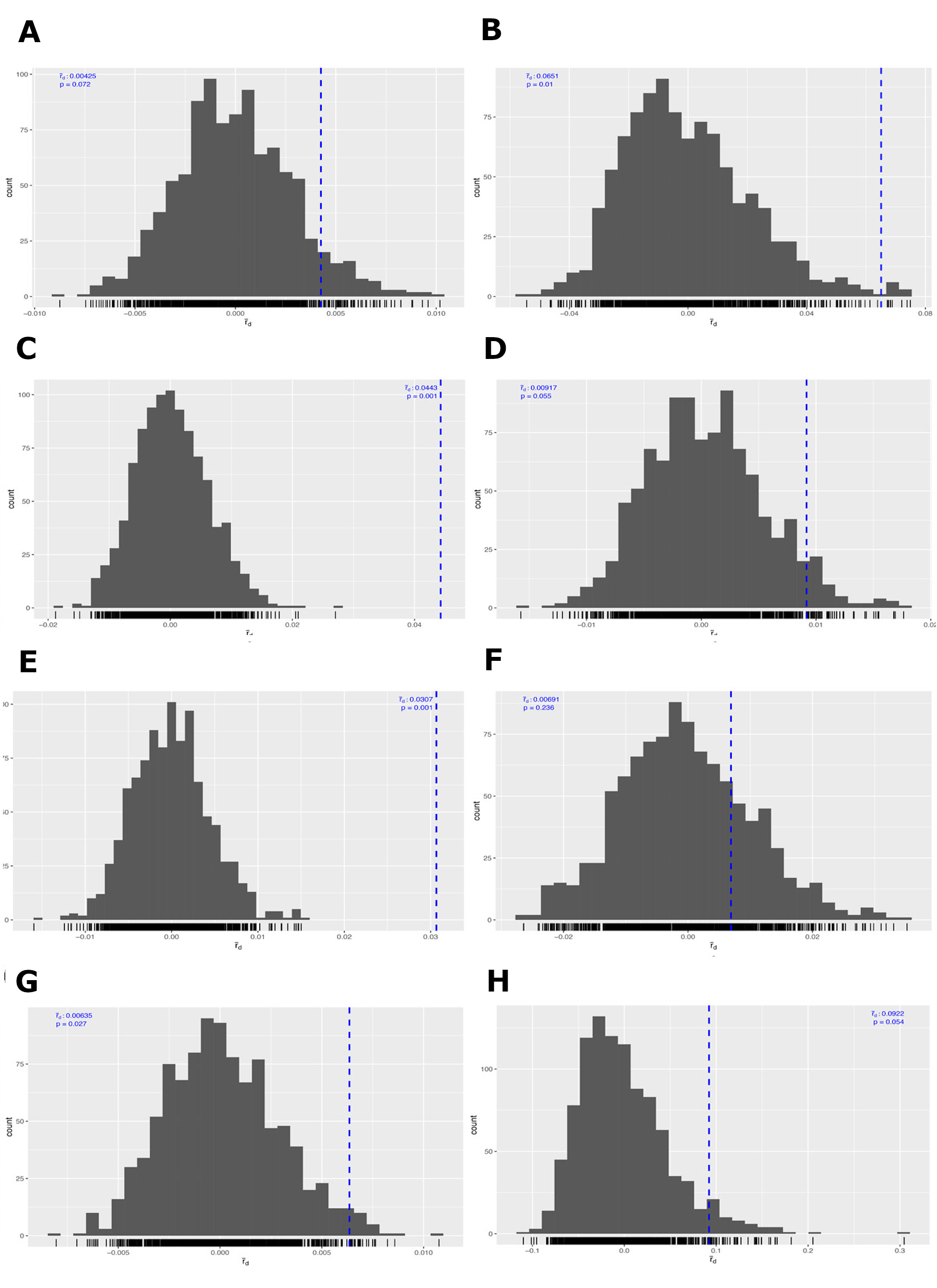


# **Supplementary Figure S3**

Tests for linkage disequilibrium between all pairs of loci in eight populations of thorn-tailed rayadito: (**A**) Fray Jorge National Park; (**B**) Cerro Santa Inés; (**C**) Cerro Manquehue; (**D**) Bariloche; (**E**) Chiloé Island; (**F**) Tierra del Fuego; (**G**) Navarino Island; (**F**) Diego Ramírez Archipelago. Correlation among loci was estimated using the standardized index of association $\overline{r}$_d_. Distributions of randomly generated values for $\overline{r}$_d_ were calculated using 1000 permutations. The dotted blue line shows the observed $\overline{r}$_d_ value.


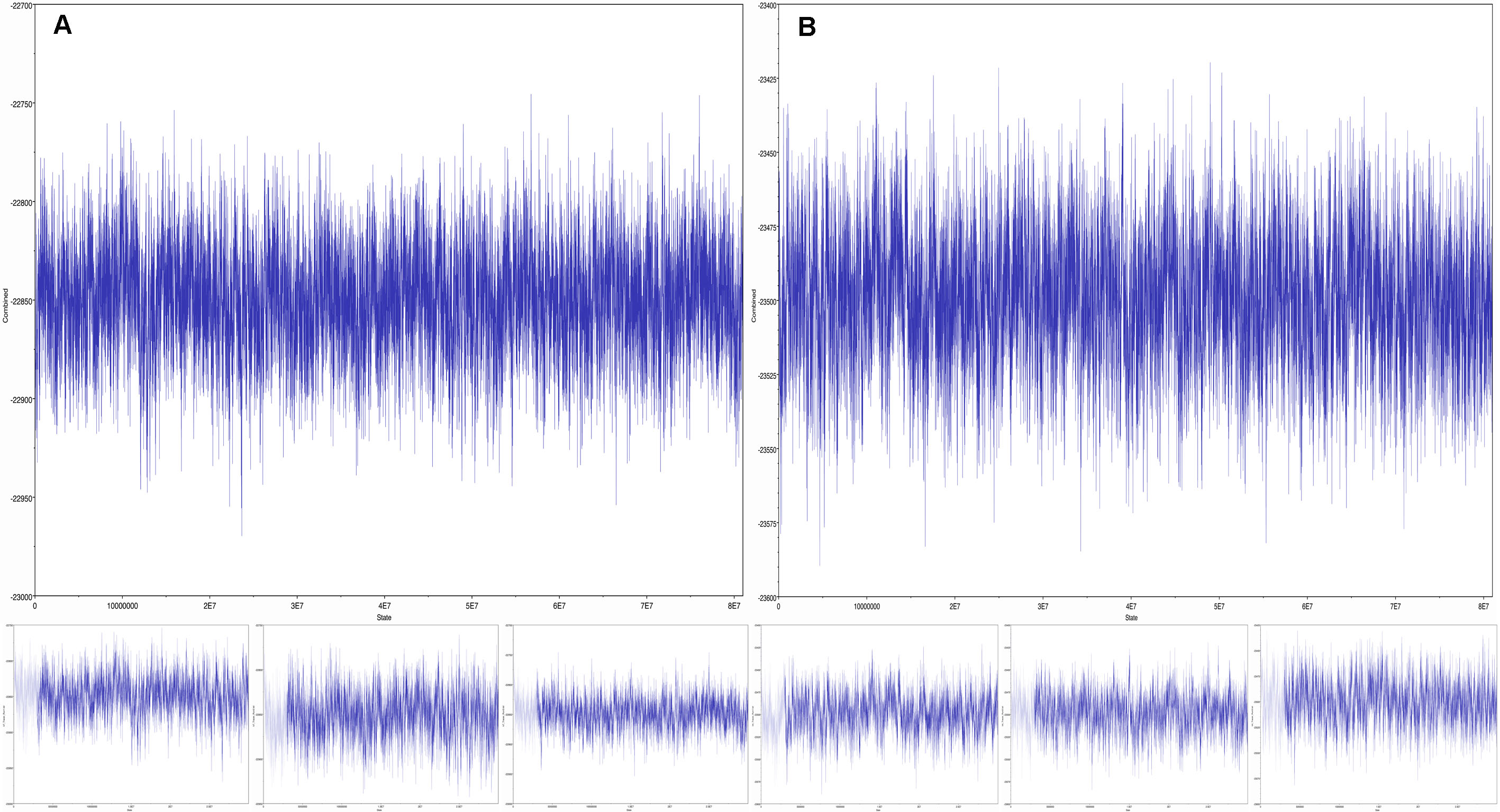


# **Supplementary Figure S4**

Inspection of the continuous parameters sampled from the Bayesian MCMC runs to assess model convergence in analyses for estimating contemporary gene flow in BayesAss 3.0.4. Diagnostics of convergence were analysed in the software Tracer 1.7.1. Big panels show the joint density (or posterior) traces from all three runs combined, whereas small panels below depict individual traces from each independent run. Shown are diagnostics for analyses using (**A**) K7 and (**B**) K3. Reported mean dispersal rates were from the second (middle small panel in A) and third (right small panel in B) runs, respectively.


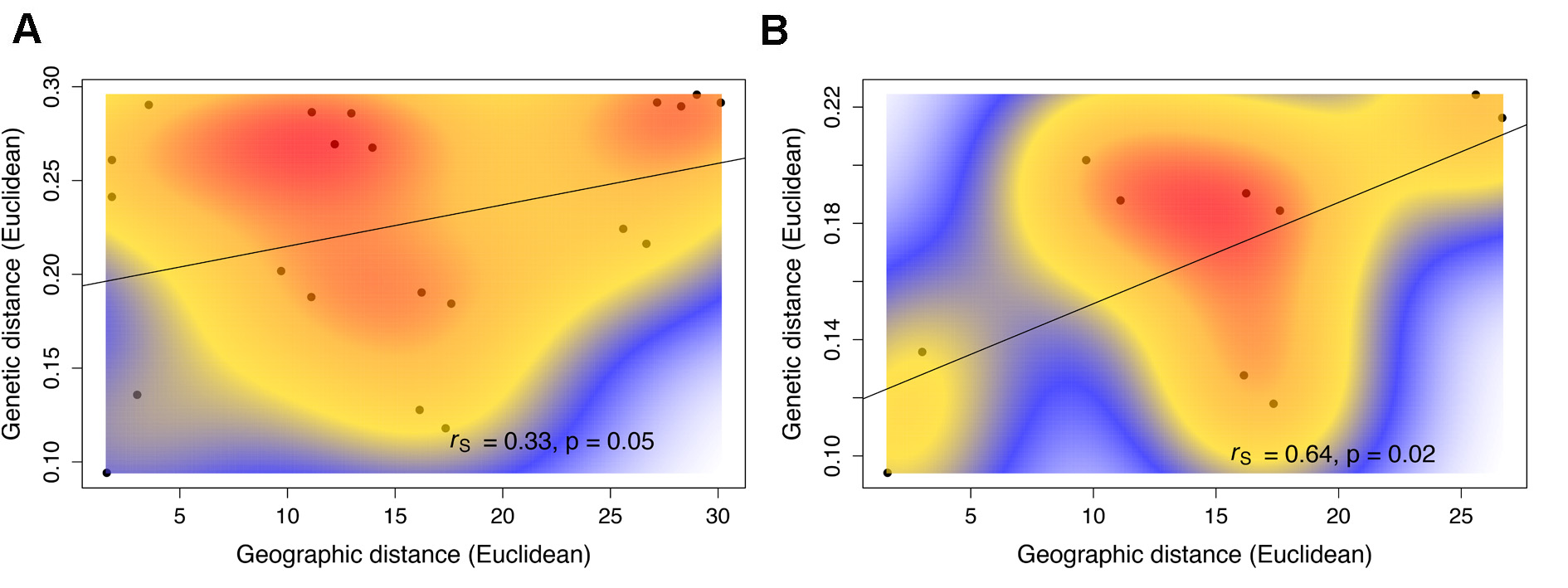


# **Supplementary Figure S5**

Plots of geographic vs. genetic distance and 2-dimensional kernel density estimation to assess isolation by distance (IBD) in populations of thorn-tailed rayadito. A gradient of low to high density is represented by a blue-red color palette. Included are the estimated correlation coefficient and a simulated p-value based on 1000 permutations to test for IBD. (**A**) Local density of points for seven continental populations of rayadito –the southernmost population from the Diego Ramírez Archipelago was not included. (**B**) Local density of points for six continental populations after additionally removing the northernmost population from Fray Jorge National Park.

**
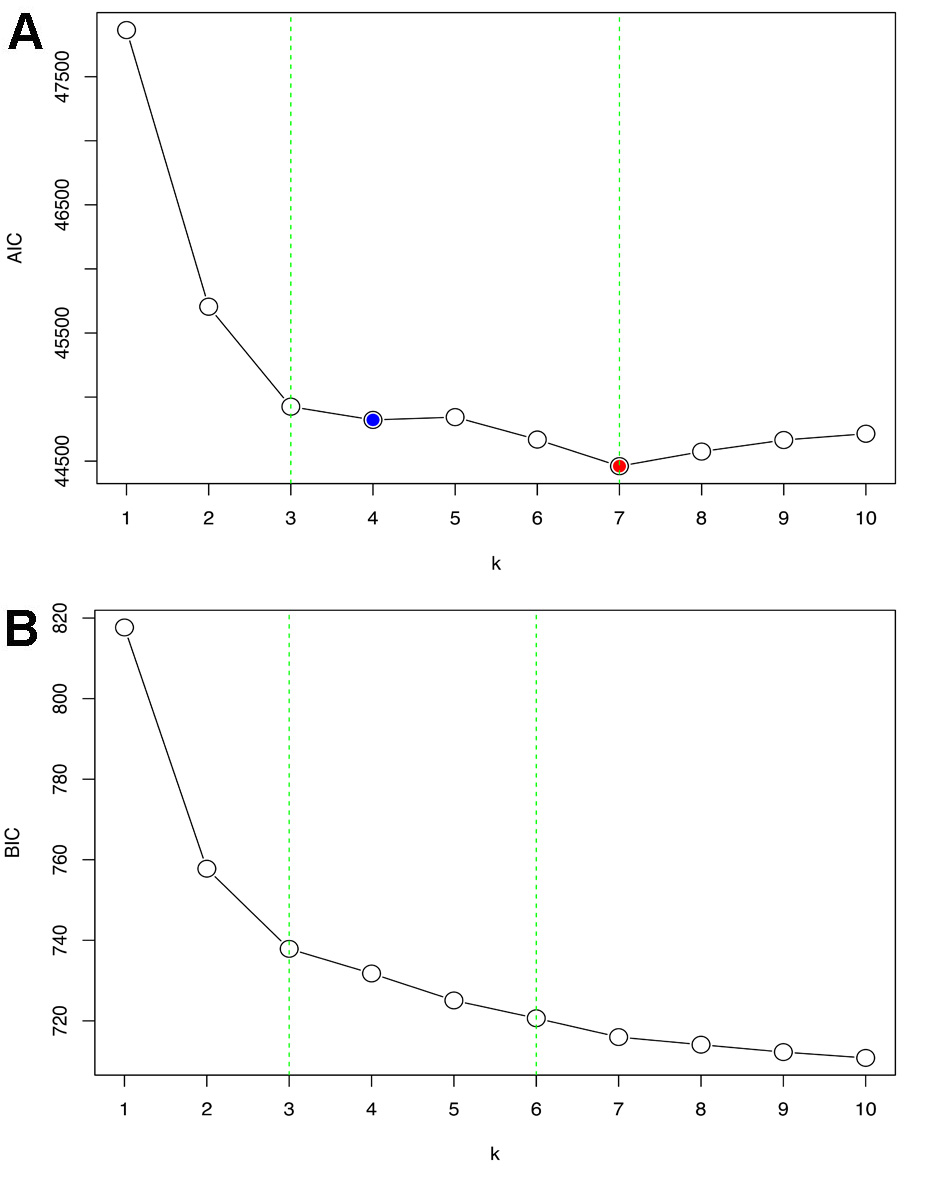
**

# **Supplementary Figure S6**

Analyses of genetic clustering used to estimate the number of cryptic genetic clusters across the breeding range of thorn-tailed rayadito. (**A**) Results from the *snapclust* analysis. The optimal number of clusters (K) was determined using the Akaike Information Criterion (AIC). The most likely number of clusters ranged between three and seven (green dotted lines). The lowest AIC value was obtained for K7 (red dot), and values plateaued between K3 and K4, slightly increasing for K5. K4 was selected as the optimal number (blue dot). (**B**) Results from a K-means clustering performed as part of a Discriminant Analysis of Principal Components (DAPC) that retained 21 PCs. The optimal number of clusters was determined using the Bayesian Information Criterion (BIC). The most likely number of clusters ranged between three and six (green dotted lines). The difference between the K6 and K7 models was small (Δ_BIC_ = 5.14).


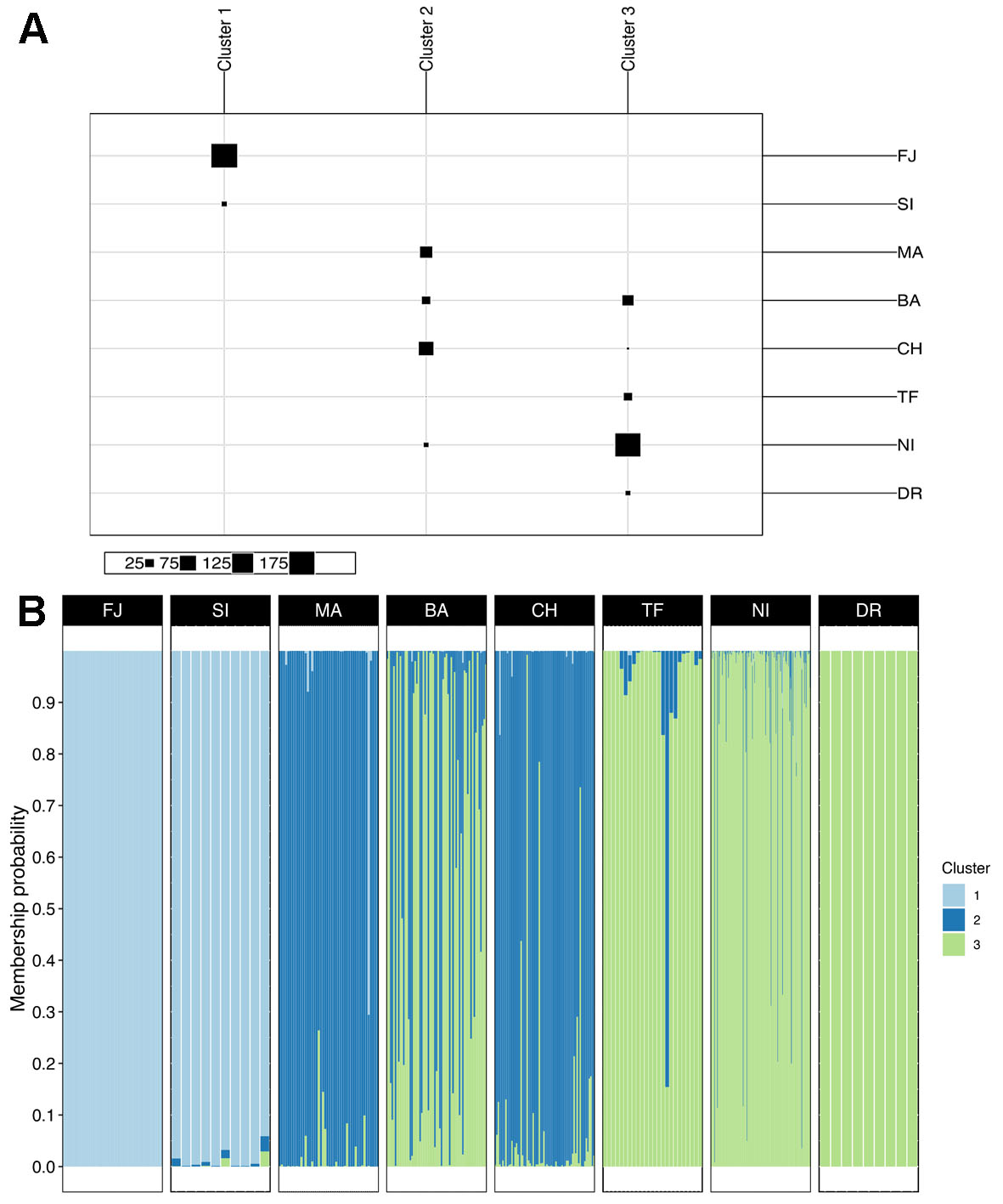


# **Supplementary Figure S7**

Genetic structure and individual assignment to genetic clusters for eight populations of thorn-tailed rayadito using 12 polymorphic microsatellite loci. FJ: Fray Jorge National Park; SI: Cerro Santa Inés; MA: Cerro Manquehue; BA: Bariloche; CH: Chiloé Island; TF: Tierra del Fuego; NI: Navarino Island; DR: Diego Ramirez Archipelago. The three genetic clusters identified by the *snapclust* method were: (1) northern cluster; (2) north-central cluster; (3) southern cluster. (**A**) Group assignments showing the distribution of sampled populations among three genetic clusters. (**B**) Assignment of individuals and probability of membership to each cluster. Each vertical bar represents an individual.

# **Appendix 3: Supplementary tables**

# **Contents:**

Supplementary Table S114

Supplementary Table S215

Supplementary Table S316

Supplementary Table S417

Supplementary Table S518

Supplementary Table S619

Supplementary Table S720

# **Supplementary Table S1**

Estimates of null allele frequency for 12 polymorphic microsatellite loci in eight populations of thorn-tailed rayadito. FJ: Fray Jorge National Park; SI: Cerro Santa Inés; MA: Cerro Manquehue; BA: Bariloche; CH: Chiloé Island; TF: Tierra del Fuego; NI: Navarino Island; DR: Diego Ramirez Archipelago. Frequencies ~0.1 are shown in bold. Negative values are obtained when the estimated frequency of null alleles was close to 0.

|  |  |  |  |  |  |  |  |  |  |
| --- | --- | --- | --- | --- | --- | --- | --- | --- | --- |
|  |  | Locality | | | | | | | |
| Locus* |  | FJ | SI | MA | BA | CH | TF | NI | DR |
| As25-14 (As2414) |  | 0.008 | -0.073 | 0.016 | 0.024 | -0.004 | 0.021 | 0.028 | ND |
| As25-10 (As2510) |  | -0.016 | 0.006 | -0.013 | -0.004 | -0.031 | 0.068 | 0.001 | ND |
| As25-8 (As2510) |  | 0.020 | -0.043 | 0.012 | 0.010 | -0.051 | -0.080 | -0.002 | ND |
| As7 (As0007) |  | 0.005 | -0.209 | **0.128** | -0.026 | 0.005 | -0.115 | -0.017 | ND |
| As18 (As0018) |  | -0.033 | -0.014 | 0.026 | -0.055 | -0.048 | 0.050 | 0.000 | ND |
| As25-1 (As2501) |  | -0.013 | -0.104 | 0.017 | 0.013 | 0.008 | -0.013 | -0.015 | ND |
| As25-5 (As2505) |  | 0.014 | 0.025 | 0.017 | 0.067 | -0.029 | -0.020 | 0.002 | ND |
| ADCYAP1 (ADC000) |  | 0.013 | -0.082 | -0.050 | 0.027 | -0.032 | 0.057 | 0.007 | ND |
| Asu15-ZEST (Asu015) | | 0.009 | 0.060 | 0.011 | -0.007 | -0.011 | -0.039 | 0.014 | ND |
| CcaTgu23 (CcaTgu) |  | 0.026 | -0.092 | -0.016 | 0.010 | -0.010 | -0.031 | -0.004 | ND |
| NED-32 (Loc032) |  | 0.001 | -0.060 | -0.015 | -0.022 | 0.002 | -0.036 | 0.015 | ND |
| Tgu05 (Tgu05D) |  | 0.001 | -0.137 | -0.005 | 0.032 | **0.129** | -0.011 | -0.008 | ND |
|  |  |  |  |  |  |  |  |  |  |

ND: not determined due to low levels of polymorphism and reduced number of genotypes in DR.

*Loci names followed the references cited in the main text. Alternative names (in parenthesis) were only used in this study and appear in other supplementary material.

# **Supplementary Table S2**

Analyses for detecting genetic signals of recent demographic changes in eight populations of thorn-tailed rayadito. FJ: Fray Jorge National Park; SI: Cerro Santa Inés; MA: Cerro Manquehue; BA: Bariloche; CH: Chiloé Island; TF: Tierra del Fuego; NI: Navarino Island; DR: Diego Ramirez Archipelago. Values of expected heterozygosity under mutation-drift equilibrium (H_eq_) were calculated using the infinite alleles model (IAM), the stepwise mutation model (SMM), and the two-phase mutation model (TPM) with varying rates of stepwise mutations –0.3, 0.5, 0.7, and 0.9. A sign test was used to test whether the number of observed loci with heterozygosity excess was different from the expected value under mutation-drift equilibrium.

|  |  |  |  |  |  |  |  |  |  |  |  |  |  |  |
| --- | --- | --- | --- | --- | --- | --- | --- | --- | --- | --- | --- | --- | --- | --- |
|  |  | IAM model | | |  | SMM model | | |  | Best TPM model* | | | | |
| Locality |  | Expected | Observed | p |  | Expected | Observed | p |  | Model | Loci fitted | Expected | Observed | p |
| FJ |  | 6.84 | 11 | 0.0117 |  | 7.06 | 5 | 0.1789 |  | 0.9 SMM | 9/12 | 7.01 | 7 | 0.6068 |
| SI |  | ND | | | | | | | | | | | | |
| MA |  | 7.07 | 10 | 0.0718 |  | 7.09 | 7 | 0.5874 |  | 0.9 SMM | 10/12 | 7.11 | 10 | 0.0756 |
| BA |  | 7.14 | 10 | 0.0774 |  | 7.07 | 1 | 0.0004 |  | 0.3 SMM | 11/12 | 7.09 | 9 | 0.2057 |
| CH |  | 7.21 | 11 | 0.0199 |  | 7.03 | 4 | 0.0703 |  | 0.9 SMM | 9/12 | 7.07 | 7 | 0.5932 |
| TF |  | 7.10 | 7 | 0.5848 |  | 7.12 | 2 | 0.0032 |  | 0.3 SMM | 11/12 | 7.10 | 5 | 0.1731 |
| NI |  | 7.06 | 11 | 0.0160 |  | 7.05 | 1 | 0.0004 |  | 0.3 SMM | 12/12 | 7.10 | 8 | 0.4150 |
| DR |  | ND | | | | | | | | | | | | |
|  |  |  |  |  |  |  |  |  |  |  |  |  |  |  |

*TPM models with different SMM rates were fitted to determine the best model for each population. A TPM model was selected as the best model whenever the observed heterozygosity values for all loci –or for the highest number observed– did not differ from H_eq_ values expected under the specified parameters. ‘Loci fitted’ shows the number of loci that did not depart from the expected values under the specified model.

# **Supplementary Table S3**

Mean (SD) dispersal rates between seven ‘continental’ populations of thorn-tailed rayadito estimated in BayesAss 3.0.4 using 573 individuals genotyped at 12 polymorphic microsatellite loci. Populations were coded as: [0] Fray Jorge National Park; [1] Cerro Santa Inés; [2] Cerro Manquehue; [3] Bariloche; [4] Chiloé Island; [5] Tierra del Fuego; [6] Navarino Island. Coefficients represent *m*, i.e. the proportion of immigrants in the population.

|  |  |  |  |  |  |  |
| --- | --- | --- | --- | --- | --- | --- |
| Population | Direction* | Mean (SD) |  | Population | Direction* | Mean (SD) |
| FJ | m[0][0]: | 0.9894(0.0043) |  | SI | m[1][0]: | 0.2060(0.0395) |
|  | m[0][1]: | 0.0017(0.0017) |  |  | m[1][1]: | 0.6862(0.0186) |
|  | m[0][2]: | 0.0018(0.0018) |  |  | m[1][2]: | 0.0241(0.0224) |
|  | m[0][3]: | 0.0018(0.0018) |  |  | m[1][3]: | 0.0198(0.0189) |
|  | m[0][4]: | 0.0018(0.0018) |  |  | m[1][4]: | 0.0192(0.0181) |
|  | m[0][5]: | 0.0018(0.0018) |  |  | m[1][5]: | 0.0197(0.0186) |
|  | m[0][6]: | 0.0018(0.0018) |  |  | m[1][6]: | 0.0249(0.0226) |
| MA | m[2][0]: | 0.0071(0.0070) |  | BA | m[3][0]: | 0.0060(0.0060) |
|  | m[2][1]: | 0.0063(0.0061) |  |  | m[3][1]: | 0.0049(0.0050) |
|  | m[2][2]: | 0.9448(0.0195) |  |  | m[3][2]: | 0.0152(0.0119) |
|  | m[2][3]: | 0.0079(0.0076) |  |  | m[3][3]: | 0.6841(0.0143) |
|  | m[2][4]: | 0.0115(0.0100) |  |  | m[3][4]: | 0.0480(0.0267) |
|  | m[2][5]: | 0.0063(0.0062) |  |  | m[3][5]: | 0.0050(0.0049) |
|  | m[2][6]: | 0.0161(0.0123) |  |  | m[3][6]: | 0.2369(0.0278) |
| CH | m[4][0]: | 0.0054(0.0053) |  | TF | m[5][0]: | 0.0107(0.0104) |
|  | m[4][1]: | 0.0046(0.0045) |  |  | m[5][1]: | 0.0109(0.0106) |
|  | m[4][2]: | 0.0234(0.0147) |  |  | m[5][2]: | 0.0110(0.0105) |
|  | m[4][3]: | 0.0068(0.0073) |  |  | m[5][3]: | 0.0113(0.0110) |
|  | m[4][4]: | 0.9408(0.0200) |  |  | m[5][4]: | 0.0123(0.0117) |
|  | m[4][5]: | 0.0046(0.0045) |  |  | m[5][5]: | 0.6774(0.0104) |
|  | m[4][6]: | 0.0144(0.0097) |  |  | m[5][6]: | 0.2665(0.0244) |
| NI | m[6][0]: | 0.0023(0.0022) |  |  |  |  |
|  | m[6][1]: | 0.0018(0.0018) |  |  |  |  |
|  | m[6][2]: | 0.0034(0.0032) |  |  |  |  |
|  | m[6][3]: | 0.0026(0.0025) |  |  |  |  |
|  | m[6][4]: | 0.0045(0.0035) |  |  |  |  |
|  | m[6][5]: | 0.0817(0.0170) |  |  |  |  |
|  | m[6][6]: | 0.9082(0.0161) |  |  |  |  |
|  |  |  |  |  |  |  |

*Examples for the notation:

[0] [0] represents the proportion of resident individuals in Fray Jorge National Park (FJNP).

[0] [1] represents the proportion of immigrants in FJNP that originated from Cerro Santa Inés.

# **Supplementary Table S4**

Mean (SD) dispersal rates between three ‘continental’ genetic clusters of thorn-tailed rayadito estimated in BayesAss 3.0.4 using 573 individuals genotyped at 12 polymorphic microsatellite loci. Genetic clusters were coded as: [0] northern cluster; [1] north-central cluster; [2] south-central cluster. Coefficients represent *m*, i.e. the proportion of immigrants in the population.

|  |  |  |
| --- | --- | --- |
| Cluster | Direction | Mean (SD) |
| Northern | m[0][0]: | 0.9946(0.0031) |
|  | m[0][1]: | 0.0018(0.0018) |
|  | m[0][2]: | 0.0018(0.0018) |
| North-central | m[1][0]: | 0.0038(0.0037) |
|  | m[1][1]: | 0.9766(0.0110) |
|  | m[1][2]: | 0.0168(0.0101) |
| South-central | m[2][0]: | 0.0018(0.0018) |
|  | m[2][1]: | 0.0221(0.0089) |
|  | m[2][2]: | 0.9749(0.0091) |
|  |  |  |

*Examples for the notation:

[0] [0] represents the proportion of resident individuals in the northern cluster (N).

[0] [1] represents the proportion of immigrants in N that originated from the north-central cluster.

# **Appendix 4: Analyses with a reduced data set**

# **Contents:**

Genetic diversity22

Range-wide genetic structure23

Contemporary gene flow26

# **1. Genetic diversity**

The plot below shows population-specific parameters of genetic diversity. As shown, genetic diversity was highest in localities at the center of the breeding range of thorn-tailed rayadito –i.e. BA and CH–, although the southern populations in TF and NI also exhibited relatively high diversity (Table 1). Genetic diversity and levels of heterozygosity were extremely low in the insular population of DR, while values for the northernmost localities were moderately low. Na: allelic richness; Na Freq > 5%: different alleles with frequency > 5%; Ne: number of effective alleles; I: Shannon’s Information Index; No. Lcomm: number of locally common alleles.

FJ: Fray Jorge National Park; SI: Cerro Santa Inés; MA: Cerro Manquehue; BA: Bariloche; CH: Chiloé Island; TF: Tierra del Fuego; NI: Navarino Island; DR: Diego Ramirez Archipelago.

# **2. Range-wide genetic structure**

*F-Statistics*

The table below shows pairwise G-Statistics used to estimate genetic differentiation between the eight sampled localities. Genetic distances ranged from -0.002 (between TF and NI) to 0.449 (between FJ and DR) when using the *G’_ST(Nei)_* index, and between -0.007 to 0.850 with the *G’’_ST_* index (Table 2). Regardless of the estimate used, pairwise G-Statistics revealed that the population in DR was strongly differentiated from the other populations (Table 2). G-Statistics also indicated that rayaditos from FJ were moderately differentiated from all populations south of SI (Table 2).

|  |  |  |  |  |  |  |  |  |
| --- | --- | --- | --- | --- | --- | --- | --- | --- |
| Locality |  |  |  |  |  |  |  |  |
|  | FJ | SI | MA | BA | CH | TF | NI | DR |
| FJ | -- | 0.221 | 0.381 | 0.422 | 0.354 | 0.393 | 0.420 | 0.850 |
| SI | 0.068 | -- | 0.247 | 0.361 | 0.316 | 0.342 | 0.380 | 0.794 |
| MA | 0.112 | 0.070 | -- | 0.199 | 0.189 | 0.219 | 0.250 | 0.688 |
| BA | 0.116 | 0.096 | 0.050 | -- | 0.163 | 0.041 | 0.046 | 0.604 |
| CH | 0.098 | 0.085 | 0.048 | 0.038 | -- | 0.214 | 0.215 | 0.705 |
| TF | 0.114 | 0.095 | 0.058 | 0.010 | 0.053 | -- | **-0.007** | 0.574 |
| NI | 0.121 | 0.106 | 0.066 | 0.011 | 0.053 | **-0.002** | -- | 0.600 |
| DR | 0.449 | 0.416 | 0.346 | 0.293 | 0.343 | 0.287 | 0.298 | -- |
|  |  |  |  |  |  |  |  |  |

Values below the diagonal correspond to the Nei’s standardized index *G’_ST(Nei)_*, while values above the diagonal were calculated using Hedrick’s standardized index corrected for small samples *G’’_ST_*_._ Excepting for the values in bold, all p < 0.001 after 1000 permutations. For values in bold: below the diagonal, p = 0.656; above the diagonal, p = 0.657.

*Principal Component Analysis*

The plot below shows the results from a Principal Component Analysis showing genetic variation in multivariate space across the breeding range of thorn-tailed rayaditos. For this analysis, we retained 41 dimensions that explained 80% of the total genetic variance. Although variation was more or less continuous, three to four groups could be discerned when plotting the first three components (19% of total variance).


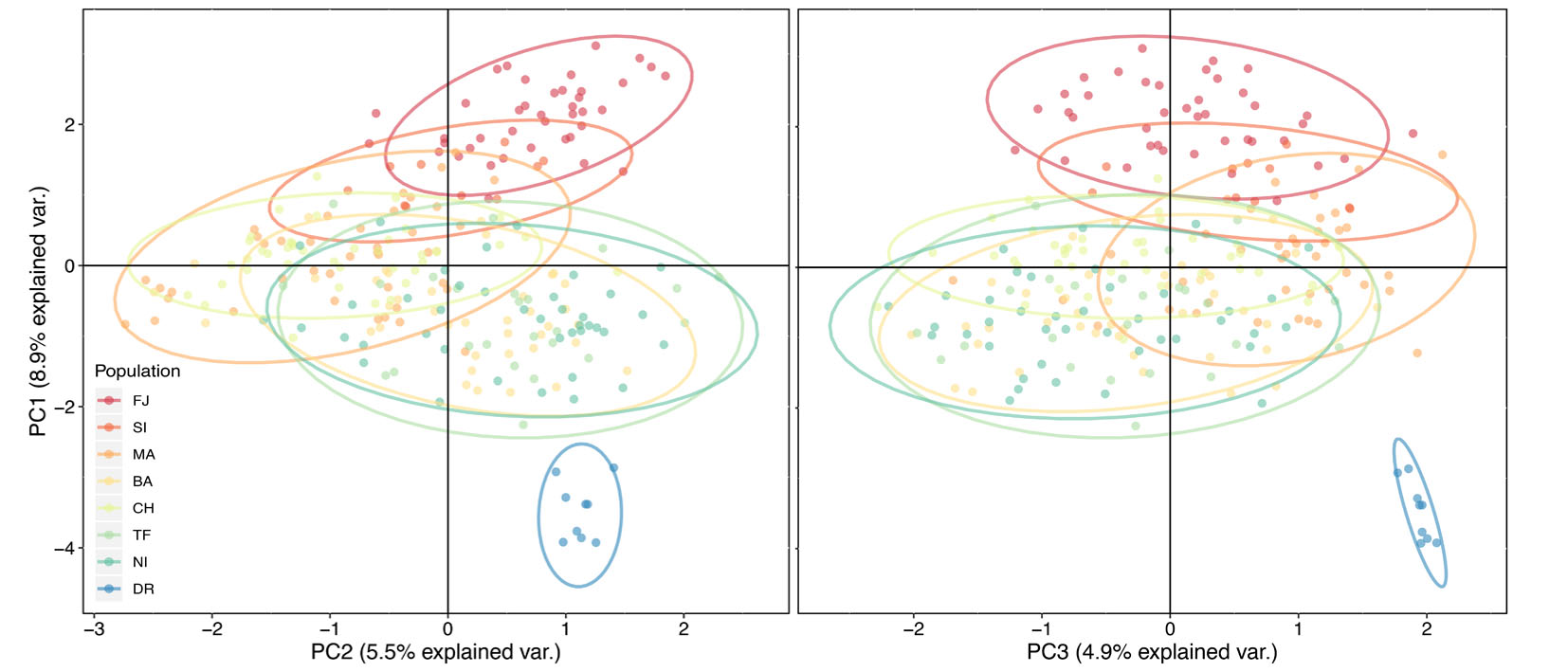


*Genetic clustering analysis*

According to the *snapclust* method, the most likely number of genetic clusters (K) in our sample ranged from three to five (green dotted lines). The lowest AIC value was obtained for K5 (red dot), and slightly increased for K6.


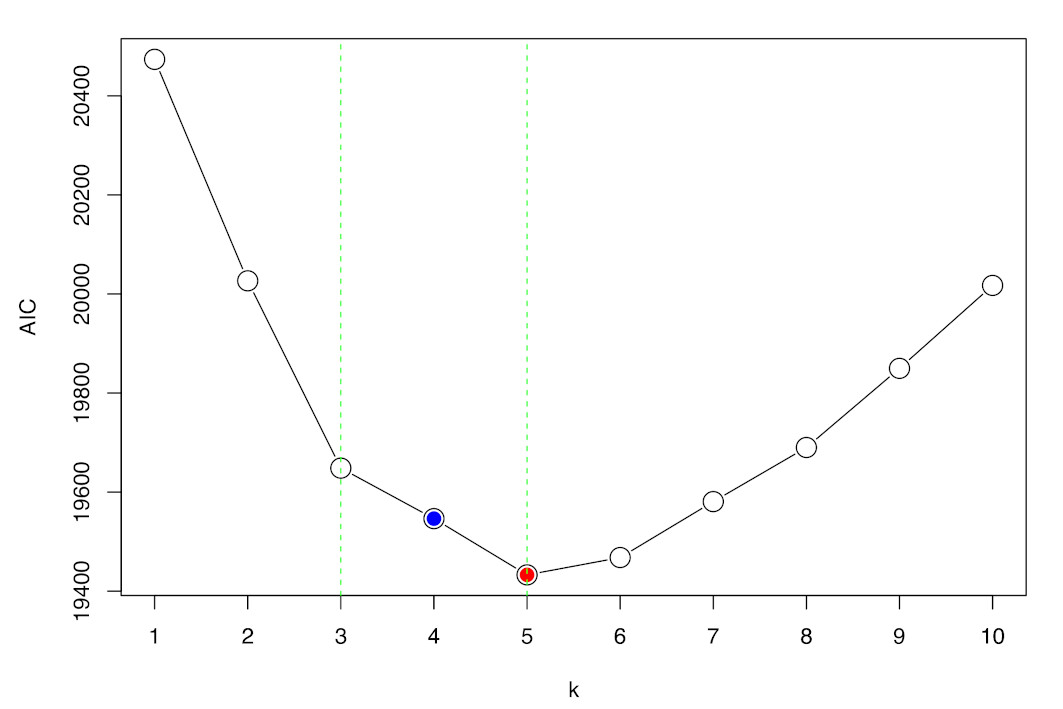


We selected K4 (blue dot) and K5 (red dot) to assess population and individual assignment.


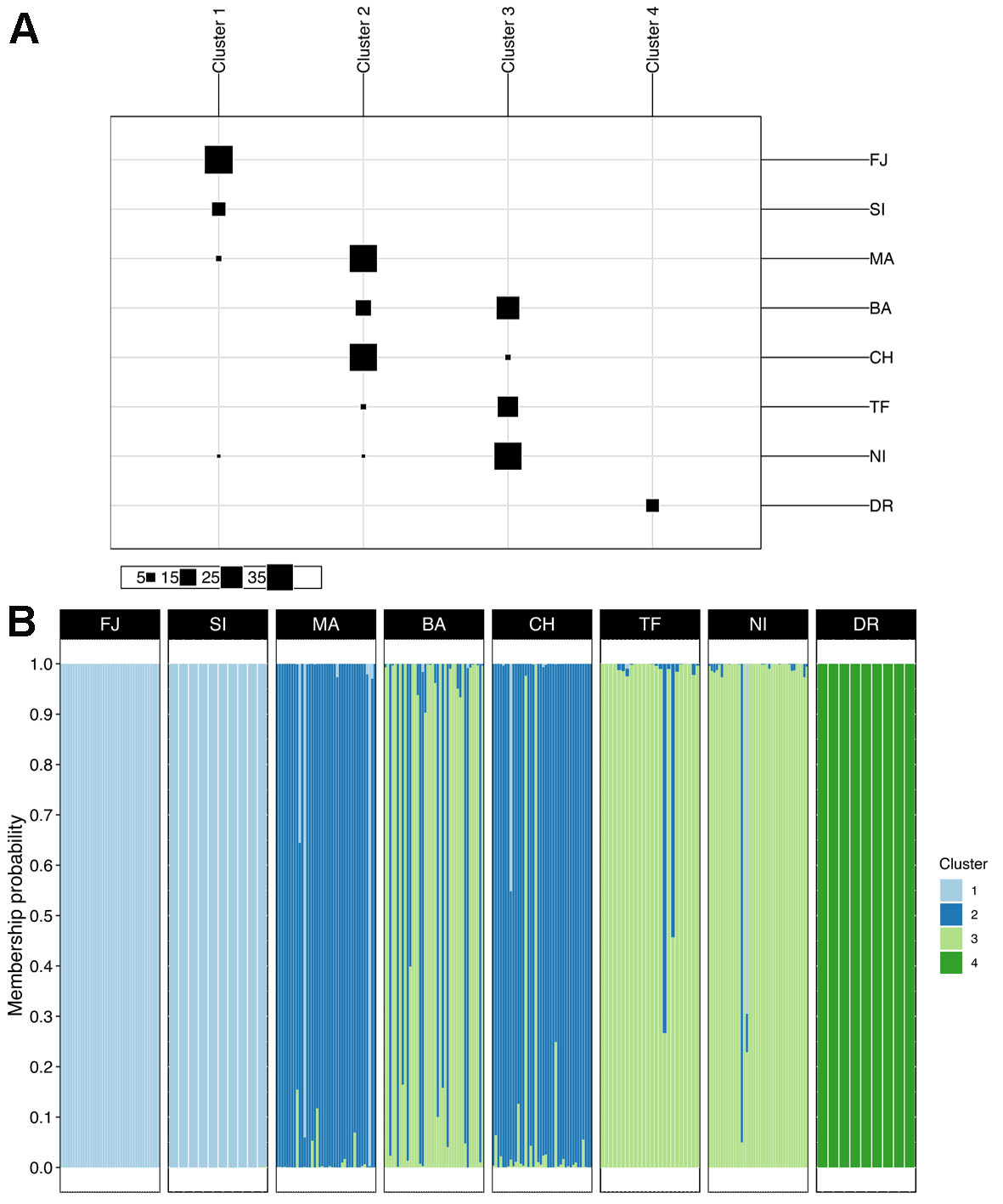


Above: genetic structure and individual assignment for eight populations of thorn-tailed rayadito. FJ: Fray Jorge National Park; SI: Cerro Santa Inés; MA: Cerro Manquehue; BA: Bariloche; CH: Chiloé Island; TF: Tierra del Fuego; NI: Navarino Island; DR: Diego Ramirez Archipelago. The four genetic clusters identified (K4) by the *snapclust* method corresponded to: (1) northern cluster; (2) north-central cluster; (3) south-central cluster; (4) Diego Ramírez population. (**A**) Group assignments showing the distribution of sampled populations among four genetic clusters. (**B**) Assignment of individuals and probability of membership to each cluster. Each vertical bar represents an individual.


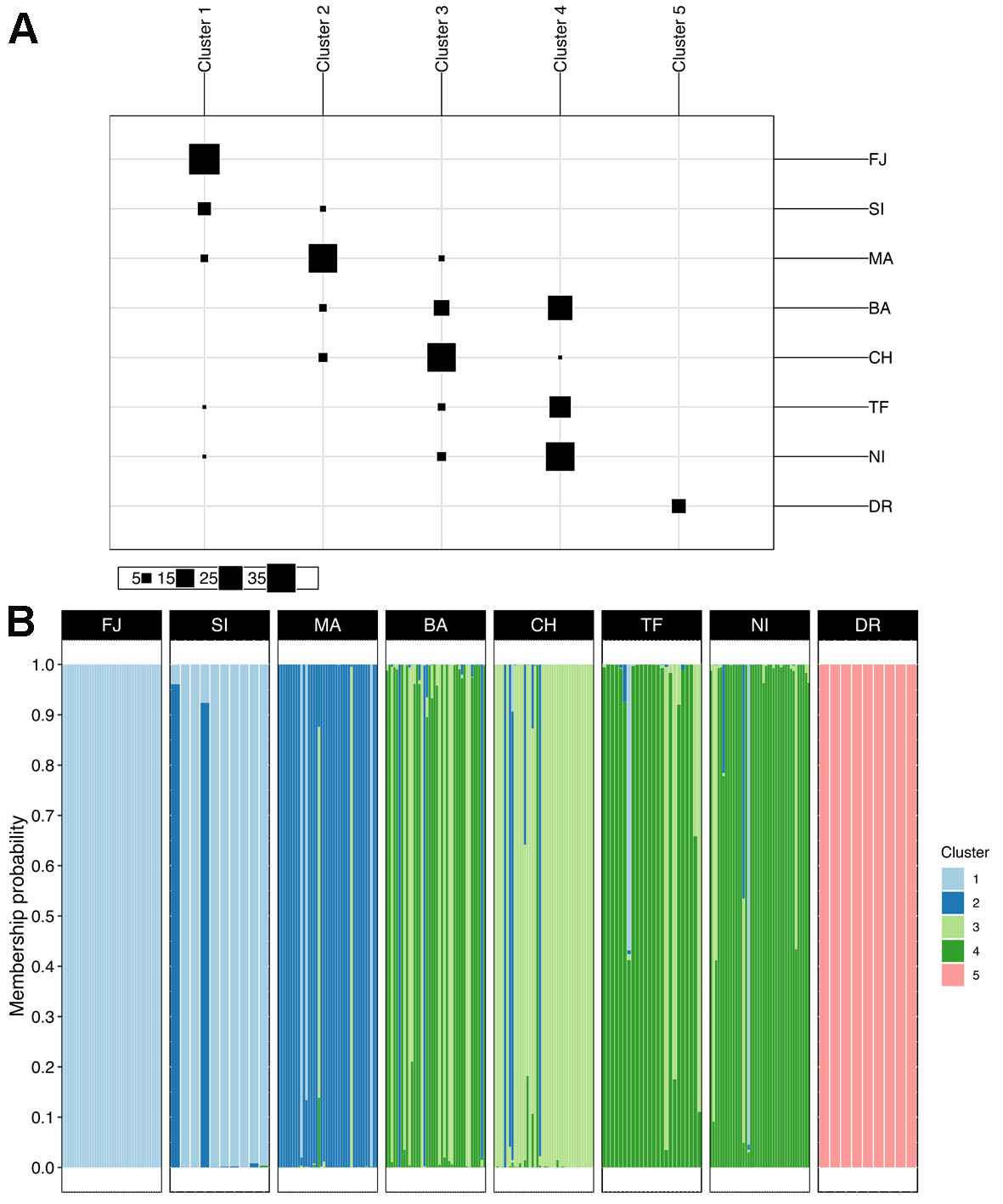


Above: genetic structure and individual assignment for eight populations of thorn-tailed rayadito. The five genetic clusters identified (K5) by the *snapclust* method corresponded to: (1) northern cluster; (2) Manquehue cluster; (3) Chiloé cluster; (4) south-central cluster; (5) Diego Ramírez population. (**A**) Group assignments showing the distribution of sampled populations among five genetic clusters. (**B**) Assignment of individuals and probability of membership to each cluster. Each vertical bar represents an individual.

# **3. Contemporary gene flow**

Mean (SD) dispersal rates between seven ‘continental’ populations of thorn-tailed rayadito estimated in BayesAss 3.0.4 using 234 individuals genotyped at 12 polymorphic microsatellite loci. Populations were coded as: [0] Fray Jorge National Park; [1] Cerro Santa Inés; [2] Cerro Manquehue; [3] Bariloche; [4] Chiloé Island; [5] Tierra del Fuego; [6] Navarino Island. Coefficients represent *m*, i.e. the proportion of immigrants in the population.

|  |  |  |  |  |  |  |
| --- | --- | --- | --- | --- | --- | --- |
| Population | Direction | Mean (SD) |  | Population | Direction | Mean (SD) |
| FJ | m[0][0]: | 0.9570(0.0161) |  | SI | m[1][0]: | 0.2154(0.0377) |
|  | m[0][1]: | 0.0071(0.0069) |  |  | m[1][1]: | 0.6860(0.0180) |
|  | m[0][2]: | 0.0074(0.0072) |  |  | m[1][2]: | 0.0197(0.0186) |
|  | m[0][3]: | 0.0072(0.0070) |  |  | m[1][3]: | 0.0198(0.0184) |
|  | m[0][4]: | 0.0072(0.0071) |  |  | m[1][4]: | 0.0196(0.0183) |
|  | m[0][5]: | 0.0071(0.0069) |  |  | m[1][5]: | 0.0199(0.0188) |
|  | m[0][6]: | 0.0072(0.0070) |  |  | m[1][6]: | 0.0196(0.0184) |
| MA | m[2][0]: | 0.0099(0.0096) |  | BA | m[3][0]: | 0.0102(0.0094) |
|  | m[2][1]: | 0.0071(0.0069) |  |  | m[3][1]: | 0.0071(0.0069) |
|  | m[2][2]: | 0.9393(0.0209) |  |  | m[3][2]: | 0.0188(0.0146) |
|  | m[2][3]: | 0.0087(0.0084) |  |  | m[3][3]: | 0.6917(0.0179) |
|  | m[2][4]: | 0.0144(0.0106) |  |  | m[3][4]: | 0.0103(0.0096) |
|  | m[2][5]: | 0.0072(0.0070) |  |  | m[3][5]: | 0.0071(0.0069) |
|  | m[2][6]: | 0.0135(0.0116) |  |  | m[3][6]: | 0.2547(0.0251) |
| CH | m[4][0]: | 0.0242(0.0166) |  | TF | m[5][0]: | 0.0108(0.0104) |
|  | m[4][1]: | 0.0070(0.0069) |  |  | m[5][1]: | 0.0107(0.0104) |
|  | m[4][2]: | 0.0198(0.0135) |  |  | m[5][2]: | 0.0113(0.0110) |
|  | **m[4][3]:** | **0.1743(0.0282)** |  |  | m[5][3]: | 0.0116(0.0112) |
|  | m[4][4]: | 0.7422(0.0243) |  |  | m[5][4]: | 0.0108(0.0105) |
|  | m[4][5]: | 0.0070(0.0069) |  |  | m[5][5]: | 0.6774(0.0103) |
|  | m[4][6]: | 0.0255(0.0169) |  |  | m[5][6]: | 0.2674(0.0238) |
| NI | m[6][0]: | 0.0125(0.0101) |  |  |  |  |
|  | m[6][1]: | 0.0071(0.0070) |  |  |  |  |
|  | m[6][2]: | 0.0106(0.0099) |  |  |  |  |
|  | m[6][3]: | 0.0112(0.0105) |  |  |  |  |
|  | m[6][4]: | 0.0102(0.0096) |  |  |  |  |
|  | m[6][5]: | 0.0937(0.0231) |  |  |  |  |
|  | m[6][6]: | 0.0855(0.0202) |  |  |  |  |
|  |  |  |  |  |  |  |

Estimates of gene flow were very similar to the values computed using the complete data set, although they tended to be slightly higher. However, changes in dispersal rates –relative to the complete data set–rarely exceeded 0.01 (4 out of 42 cases), and patterns of demographic interactions among populations remained as shown in Fig. 3 (see main text). Only the estimate of gene flow from BA to CH noticeably increased compared to the complete data set (17.4% versus 0.6%; **in bold**).

Mean (SD) dispersal rates between three ‘continental’ genetic clusters of thorn-tailed rayadito estimated in BayesAss 3.0.4 using 90 individuals genotyped at 12 polymorphic microsatellite loci. Genetic clusters were coded as: [0] northern cluster; [1] north-central cluster; [2] south-central cluster. Coefficients represent *m*, i.e. the proportion of immigrants in the population.

|  |  |  |
| --- | --- | --- |
| Cluster | Direction | Mean (SD) |
| Northern | m[0][0]: | 0.9771(0.0127) |
|  | m[0][1]: | 0.0077(0.0075) |
|  | m[0][2]: | 0.0077(0.0075) |
| North-central | m[1][0]: | 0.0224(0.0167) |
|  | m[1][1]: | 0.8381(0.0464) |
|  | **m[1][2]:** | **0.1316(0.0448)** |
| South-central | m[2][0]: | 0.0120(0.0106) |
|  | m[2][1]: | 0.0373(0.0241) |
|  | m[2][2]: | 0.9425(0.0264) |
|  |  |  |

Estimates of gene flow were very similar to the values computed using the complete data set, although they tended to be slightly higher. With the exception of one case, changes in dispersal rates –relative to the complete data set– did not exceed 0.02. Overall, patterns of demographic interactions among clusters remained as shown in Fig. 3 (see main text). Only the estimate of gene flow from the south-central to the north-central cluster noticeably increased compared to the complete data set (13.2% versus 1.7%; **in bold**).

# **References:**

Benjamini, Y. & Hochberg, Y. Controlling the false discovery rate: a practical and powerful approach to multiple testing. *J. R. Statist. Soc. B.* **57**, 289­–300.

Beugin, M-P., Gayet, T., Pontier, D., Devillar, S., & Jombart, T. A fast likelihood solution to the genetic clustering problem. *Methods Ecol. Evol.* **9**, 1006–1016 (2018).

Botero-Delgadillo, E. *et al*. Variation in fine-scale genetic structure and local dispersal patterns between peripheral populations of a South American passerine bird. *Ecol. Evol* **7**, 8363–8378 (2017a).

Botero-Delgadillo, E., Orellana, N., Serrano, D., Poblete, Y., & Vásquez, R. A. Interpopulation variation in nest architecture in a secondary cavity-nesting bird suggests site-specific strategies to cope with heat loss and humidity. *Auk* **134**, 281–294 (2017b).

Botero-Delgadillo, E. *et al*. Ecological and social correlates of natal dispersal in female and male Thorn-tailed Rayadito (*Aphrastura spinicauda*) in a naturally isolated and fragmented habitat. *Auk* **136**, ukz016 (2019).

Cornuet, J-M. & Luikart, G. Description and power analysis of two tests for detecting recent population bottlenecks from allele frequency data. *Genetics* **144**, 2001–2014 (1996).

Di Rienzo *et al*. Mutational processes of simple sequence repeat loci in human populations. *Proc. Natl. Acad. Sci. USA* **91**, 3166–3170 (1994).

Do, C., Waples, R. S., Peel, D., Macbeth, G. M., Tillet, B. J., & Ovenden, J. R. NeEstimator V2: re-implementation of software for the estimation of contemporary effective population size (*N_e_*) from genetic data. *Mol. Ecol. Res.* **14**, 209–214 (2014).

Espíndola-Hernández, P., Castaño-Villa, G. J., Vásquez, R. A., & Quirici, V. Sex-specific provisioning of nutritious food items in relation to brood sex ratios in a non-dimorphic bird. Behav. Ecol. Sociobiol. **71**,65 (2017).

Faubet, P., Waples, R. S., & Gaggiotti, O. E. Evaluating the performance of a multilocus Bayesian method for the estimation of migration rates. *Mol. Ecol.* **16**, 1149–1166 (2007).

Goudet, J. & Jombart, T. hierfstat: estimation and tests of hierarchical F-Statistics. *R package version 0.04-22* <https://CRAN.R-project.org/package=hierfstat> (2015).

Hedrick, P. W. A standardized genetic differentiation measure. *Evolution* **59**, 1633–1638 (2005).

Jombart, T. adegenet: A R package for the multivariate analysis of genetic markers. *Bioinformatics* **24**, 1403–1405 (2008).

Jombart, T., Devillard, S., & Balloux, F. Discrimnant analysis of principal components: A new method for the analysis of genetically structured populations. *BMC Genetics* **11**, 94 (2010).

Kamvar, Z. N., Tabima, J. F., Grünwald, N. J. Poppr: an R package for genetic analysis of populations with clonal, partially clonal, and/or sexual reproduction. *PeerJ* **2**, e281 (2014).

Luikart, G. & Cornuet, J-M. Empirical evaluation of a test for identifying recently bottlenecked populations from allele frequency data. *Conserv. Biol*. **12**, 228–237 (1998).

Meirmans, P. G. & Hedrick, P. W. Assessing populations structure: Fst and related measures. *Mol. Ecol*. **11**, 5–18 (2011).

Moreno, J., Merino, S., Vásquez, R. A., & Armesto, J. J. Breeding biology of the Thorn-tailed Rayadito (Furnariidae) in south-temperate rainforests of Chile. *Condor* **107**, 69–77, (2005).

Moreno, J., Merino, S., Lobato, E., Rodríguez-Gironés, M. A., & Vásquez, R. A. Sexual dimorphism and parental roles in the Thorn-tailed Rayadito (Furnariidae). *Condor* **109**, 312–320 (2007).

Nei, M. Molecular Evolutionary Genetics (Columbia University Press, 1987).

Ippi, S., van Dongen, W. F. D., Lazzoni, I., & Vásquez, R. A. Shared territorial defence in the suboscine Thorn-tailed Rayadito (*Aphrastura spinicauda*). *Emu* **117**, 97–102 (2017).

Peakall, R. & Smouse, P. E. GenAlEx 6.5: genetic analysis in Excel. Population genetic software for teaching and research –an update. *Bioinformatics* **28**, 2537–2539 (2012).

Patterson, N., Price, A. L., & Reich, D. Population structure and eigenanalysis. *PLoS Genetics* **2**, 2071–2093 (2006).

Putman, A. I. & Carbone, I. Challenges in analysis and interpretation of microsatellite data for population genetic studies. *Ecol. Evol*. **4**, 4399–4428 (2014).

Piry, S. *et al*. GeneClass2: A software for genetic assignment and first-generation migrant detection. *J. Heredity* **95**, 536–539 (2004).

Quirici, V. *et al*. Baseline corticosterone and stress response in the Thorn-tailed Rayadito (*Aphrastura spinicauda*) along a latitudinal gradient. *Gen. Comp. Endocr.* **198**, 39–46 (2014).

Quirici, V., Guerrero, C. J., Krause, J. S., Wingfield, J. C., & Vásquez, R. A. The relationship of telomere length to baseline corticosterone levels in nestlings of an altricial passerine bird in natural populations. *Frontiers Zool.* **13**,1 (2016).

R Core Team. R: a language and environment for statistical computing, version 3.5.2. *R Foundation for Statistical Computing* <http://www.R.project.org> (2018).

Rambaut, A., Drummond, A. J., Xie, D., Baele, G., & Suchard, M. A. Posterior summarisation in Bayesian phylogenetics using Tracer 1.7. *Syst. Biol.* **67**, 901–904 (2018).

Shriver, M. D., Jin, L., Chakraborty, R., & Boerwinkle, E. VNTR allele frequency distributions under the stepwise mutation model –a computer simulation approach. *Genetics* **134**, 983–993 (1993).

Wilson, G. A. & Rannala, B. Bayesian inference of recent migration rates using multilocus genotypes. *Genetics* **163**, 1177–1191 (2003).

Yáñez, D. I., Quirici, V., Castaño-Villa, G. J., Poulin, E., & Vásquez, R. A. Isolation and Characterisation of Eight Microsatellite Markers of the Thorn-Tailed Rayadito *Aphrastura spinicauda*. *Ardeola* **62**,179–183 (2015).
